# Zhilong Huoxue Tongyu capsule improves myocardial ischemia/reperfusion injury via the PI3K/AKT/Nrf2 axis

**DOI:** 10.1371/journal.pone.0302650

**Published:** 2024-04-30

**Authors:** Xiaoping Zhao, Fang Yang, Hao Wu, Zhongcai Fan, Gang Wei, Yuan Zou, Jinyi Xue, Mengnan Liu, Gong Chen

**Affiliations:** 1 Department of Cardiology, The Affiliated Hospital of Southwest Medical University, Luzhou, Sichuan, China; 2 National Traditional Chinese Medicine Clinical Research Base and Department of Cardiovascular Medicine, The Affiliated Traditional Chinese Medicine Hospital of Southwest Medical University, Luzhou, Sichuan, China; 3 Institute of Integrated Chinese and Western Medicine, Southwest Medical University, Luzhou, Sichuan, China; University of California, Davis, UNITED STATES

## Abstract

**Introduction:**

Zhilong Huoxue Tongyu Capsule (ZL) is a Chinese medicine used for the treatment of cardio-cerebral diseases. However, the pharmacological mechanisms underlying its regulation of myocardial ischemia/reperfusion injury (MI/RI) remain unclear.

**Purpose:**

This study aims to investigate the effects and mechanisms of ZL on MI/RI in mice.

**Materials and methods:**

C57BL/6J mice were randomly assigned to four groups: Sham group, I/R group, ZL group, and ZLY group. The MI/RI mouse model was established by ligation of the left anterior descending coronary artery for 30 minutes, followed by reperfusion for 120 minutes to restore blood perfusion. Cardiac function was evaluated using cardiac ultrasound. Histopathological changes and myocardial infarction area were assessed using Hematoxylin and eosin (H&E) staining and triphenyltetrazolium chloride (TTC) staining. The changes in oxidative stress- and ferroptosis-related markers were detected. RT-qPCR, Western blot, and ELISA were conducted to further explore the mechanism of ZL in improving MI/RI.

**Results:**

Our findings demonstrated that ZL exerted a protective effect against MI/RI by inhibiting ferroptosis, evidenced by the upregulation of antioxidant enzymes such as GSH and GPX4, coupled with the downregulation of ACSL4, a pro-ferroptosis factor. Furthermore, ZL positively impacted the PI3K/AKT/Nrf2 pathway by promoting ATPase activities and enhancing the relative protein expression of its components. Notably, the administration of a PI3K/AKT inhibitor reversed the antioxidant and anti-ferroptosis effects of ZL to some extent, suggesting a potential role for this pathway in mediating ZL’s protective effects.

**Conclusions:**

ZL protects against MI/RI-induced ferroptosis by modulating the PI3K/AKT signaling pathway, leading to increased Nrf2 expression and activation of the HO-1/GPX4 pathway. These findings shed light on the potential therapeutic mechanisms of ZL in the context of cardiovascular diseases.

## Introduction

Acute Myocardial Infarction (AMI) represents a critical condition within the spectrum of cardiovascular diseases. Timely and effective reperfusion therapy is crucial to salvage the surviving myocardium and alleviate complications [1, 2]. However, the reperfusion process itself can paradoxically exacerbate myocardial tissue injury, leading to Myocardial Ischemia/Reperfusion Injury (MI/RI) [3]. Given the prevalence of MI/RI, its contribution to increased myocardial infarct size and poor prognosis, and the current lack of highly reliable therapeutic strategies specifically targeting MI/RI damage in clinical practice, the prevention and reduction of MI/RI have become pivotal areas of focus in both clinical and basic research.

The pathophysiological mechanisms underlying MI/RI are complex and involve multiple factors. Research has revealed that ferroptosis plays a critical role in the development and progression of MI/RI [4–6]. Suzuki et al. [7, 8] demonstrated that the transcription factor Nuclear Factor Erythroid 2-Related Factor 2 (Nrf2) is closely associated with oxidative stress and ferroptosis. It is now understood that Nrf2 promotes the transcription of downstream target genes, including heme oxygenase-1 (HO-1) and glutathione peroxidase 4 (GPX4), both of which play crucial roles in resistance to ferroptosis [9]. Recent research has highlighted the pivotal role of the phosphoinositide-3-kinase/protein kinase B (PI3K/AKT) pathway in ischemia-reperfusion and oxidative stress-induced diseases. It has been found that the phosphorylation of PI3K/AKT could promote Nrf2 expression [10, 11].

In recent years, there has been a growing interest in harnessing traditional Chinese medicine (TCM) for the prevention and treatment of cardiovascular diseases. ZL is a Chinese medicine used for the treatment of cardio-cerebral diseases, comprised of *Astragalus*, *Pheretima*, *Sargentodoxae Caulis*, *Cassia Twig*, and *Hirudo* in a ratio of 8:4:4:3:1 [12]. Previous studies have shown that ZL exerts anti-inflammatory, anti-apoptotic, and pro-angiogenic effects through multi-component, multi-pathway, and multi-target actions for treating these diseases [13]. Importantly, studies using the maximum tolerated dose (81.6 g/kg/d) in mice observed no obvious effects on physiological function or long-term toxicity [14]. Furthermore, ZL was found to be effective in attenuating cerebral ischemia/reperfusion injury [12]. Based on these findings, we further investigated the influence of ZL on MI/RI-induced ferroptosis through its potential regulation of the PI3K/AKT/Nrf2 signaling pathway, aiming to elucidate the mechanisms underlying its cardioprotective effects.

## Materials and methods

### Drugs and reagents

Zhilong Huoxue Tongyu capsule (Med-drug permit no. Chuan Z20070528; Patent number 200810147774.1) was provided by the Preparation Room for TCM at the Affiliated Hospital of TCM, Southwest Medical University. The specific information of antibodies (p-PI3K, PI3K, p-AKT, AKT, Nrf2, HO-1, GPX4, ACSL4, GAPDH) is presented in S1 Table. RNA extraction, reverse transcription, and reaction reagents were purchased from TOYOBO (Shanghai) Biotech Co., Ltd. The BCA protein concentration assay kit and protein lysis buffer were purchased from Beyotime Biotechnology Co., Ltd. The lactate dehydrogenase (LDH) kit, Creatine kinase-MB isoenzyme (CK-MB) kit, malondialdehyde (MDA) kit, superoxide dismutase (SOD) kit, reactive oxygen species (ROS) kit, and ELISA kit were purchased from Jiancheng (Nanjing) Biotech Co., Ltd.

### Ethics statement and animals

Pathogen-free healthy mice were purchased from Chengdu Dashuo Experimental Animal Co. (Certification No. SYXK Chuan 2020–030) and maintained in a feeding environment with a temperature of 19–22°, humidity of 40–60%, and a standard light-dark cycle. All experimental procedures were performed in accordance with the National Institute of Health Guide for the Care and Use of Laboratory Animals, and the experimental plan was approved by the Animal Research Committee of Southwest Medical University (Approval Number. SWMU20230095).

### Experimental grouping

Forty SPF-grade C57BL/6J mice were randomly divided into four groups: Sham, I/R, ZL pre-treated (ZL), and ZL pre-treated + LY294002 (ZLY) groups. The ZL and ZLY groups received Zhilong Huoxue Tongyu Capsules (6.24 g/kg/d) through gavage for 14 consecutive days before modeling. LY294002 (0.3 mg/kg) was intraperitoneally injected into the ZLY group 30 minutes before modeling.

### Animal model

Mice underwent a 12-hour fasting period before surgery. Anesthesia was induced using 3% isoflurane, and mice were positioned supine on a temperature-controlled heating pad connected to an electrocardiogram (ECG) monitor for continuous monitoring. The surgical area was prepared by making a skin incision, opening the thoracic cavity, and exposing the pericardium. Mice were then carefully transferred to a microscope for further manipulations, where the left anterior descending coronary artery was ligated approximately 2 mm below the lower edge of the left auricle. The Sham group underwent a similar procedure without coronary artery ligation. Successful induction of ischemia was confirmed by the presence of a ventricular ischemic pale area and an ECG showing ST segment bow-back elevation or depression of 0.1 mV. After 30 minutes of ligation, reperfusion for 120 minutes was initiated, marked by a 1/2 decrease in the previously elevated ST segment. Following successful reperfusion, accumulated blood in the chest cavity was cleared, and the chest wall was meticulously sutured layer by layer. The ventilator was discontinued after spontaneous breathing was restored.

### Sample collection

After 120 minutes of reperfusion, left ventricular ejection fraction (EF, %) and shortening fraction (FS, %) were evaluated using cardiac ultrasound (VisualSonics, Canada). Blood samples were collected via retro-orbital bleeding and centrifuged at 4°C, 3000 rpm for 5 minutes to collect supernatant serum. Mice anesthetized with 10% isoflurane were put into deep anesthesia to avoid suffering, and then the spinal cord was isolated leading to death, and myocardial tissue was collected from each group. The myocardial tissue was divided into three sections: one fixed in 4% paraformaldehyde, another used for TTC staining, and the remainder frozen at -80°C for RNA and protein extraction.

### Biochemical assays

Serum LDH and CK-MB levels were measured using ELISA kits. Myocardial tissue homogenates were prepared, and protein concentrations were determined. Subsequently, Fe^2+^, ROS, MDA, GSH, and SOD levels were measured in the homogenates using appropriate kits.

### Infarct size assessment

The myocardial infarct sizes were measured using triphenyltetrazolium chloride (TTC, Solarbio, China) staining. Hearts were frozen at -20°C for 20 minutes. Then, the tissue was cut into 5 pieces, 1–2 mm thick, and incubated in 2% TTC solution for 30 minutes at 37°C. Tissue sections were then fixed in 4% paraformaldehyde solution for 24 hours. Images were collected using a digital camera. The infarcted size was calculated using ImageJ software: Myocardial infarct size = sum of infarct area / whole heart area.

### Pathological staining

Following fixation, tissues were embedded in paraffin, sectioned, and stained with H&E following the kit instructions. Stained sections were then observed under a bright field microscope.

### Immunohistochemistry

Immunohistochemistry was employed to assess the expression of P-PI3K and P-AKT in myocardial tissue. Paraffin sections were initially blocked, followed by incubation with normal goat serum at room temperature for 10 minutes. Subsequently, the sections were incubated with primary antibodies: p-PI3K (dilution 1:100) and p-AKT (dilution 1:50) at 4°C overnight. The following day, the slides were incubated with a secondary antibody (dilution 1:100) followed by a DAB reaction for color development. Stained slides were then observed under a bright field microscope, and images were captured. Quantitative analysis of positively stained areas was conducted using Image J software.

### Immunofluorescence

Paraffin sections were dewaxed, followed by antigen retrieval and incubation with 3% hydrogen peroxide to inactivate endogenous enzymes. After washing, sections were blocked with goat serum and incubated with Nrf2 primary antibody (dilution 1:100) at 4°C overnight. The slides were then washed with PBS and incubated with a fluorescent secondary antibody (HRP-labeled goat anti-mouse IgG, dilution 1:100) for 30 minutes at room temperature in the dark. Following another wash, incubation with anti-HO-1 primary antibody was performed using the same protocol. A fluorescent (FITC)-labeled goat anti-IgG secondary antibody (dilution 1:500) was used. Finally, the slides were stained with DAPI for nuclear counterstaining. Images were captured using an upright fluorescence microscope (BA210LED, Nikon, Japan). Mean gray value was analyzed using ImageJ software.

### RT-qPCR

Total RNA was extracted from cardiac tissue using the TOYOBO kit (Shanghai, China) following the manufacturer’s instructions. A fluorescence quantitative PCR instrument (LightCycler 480II, Roche, Switzerland) was employed for routine melting curve analysis and Ct value determination after reverse transcription. β-actin served as the internal reference gene. The relative expression level of the target mRNA was quantified using the 2^-ΔΔCt^ method. The primer sequences for Nrf2, HO-1, ACSL4, and GPX4 are provided in S2 Table.

### Western blot analysis

Protein extraction from the cardiac tissue of mice was conducted using standard procedures. Protein concentration was determined using the BCA method. The protein samples were then mixed with loading buffer and denatured at 100°C for 10 minutes. SDS-bisacrylamide gel electrophoresis was employed to separate the proteins, followed by transfer onto a PVDF membrane. The membrane was blocked with TBST solution containing 5% bovine serum albumin. Primary antibodies (p-PI3K (1:1000), PI3K (1:1000), p-AKT (1:1000), AKT (1:1000), Nrf2 (1:1000), HO-1 (1:2000), GPX4 (1:2000), ACSL4 (1:1000), and GAPDH (1:5000)) were added and incubated at 4°C overnight. Following washes the next day, the secondary antibody (1:10000) was applied and incubated at room temperature for 2 hours. After another round of washing, a Chemiluminescence imaging system was employed to capture images. Band intensities were analyzed using ImageJ software for grayscale value quantification.

### Statistical analysis

GraphPad Prism Ver 9.0 was used for statistical analysis. Data were expressed as mean ± standard error of the mean (SEM). The appropriate one-way or two-way analysis of variance (ANOVA) was used, followed by the LSD t-test for pairwise comparisons between groups. A P-value of less than 0.05 was considered statistically significant.

## Results

### Cardioprotective effect of ZL against MI/RI

**Fig 1:**
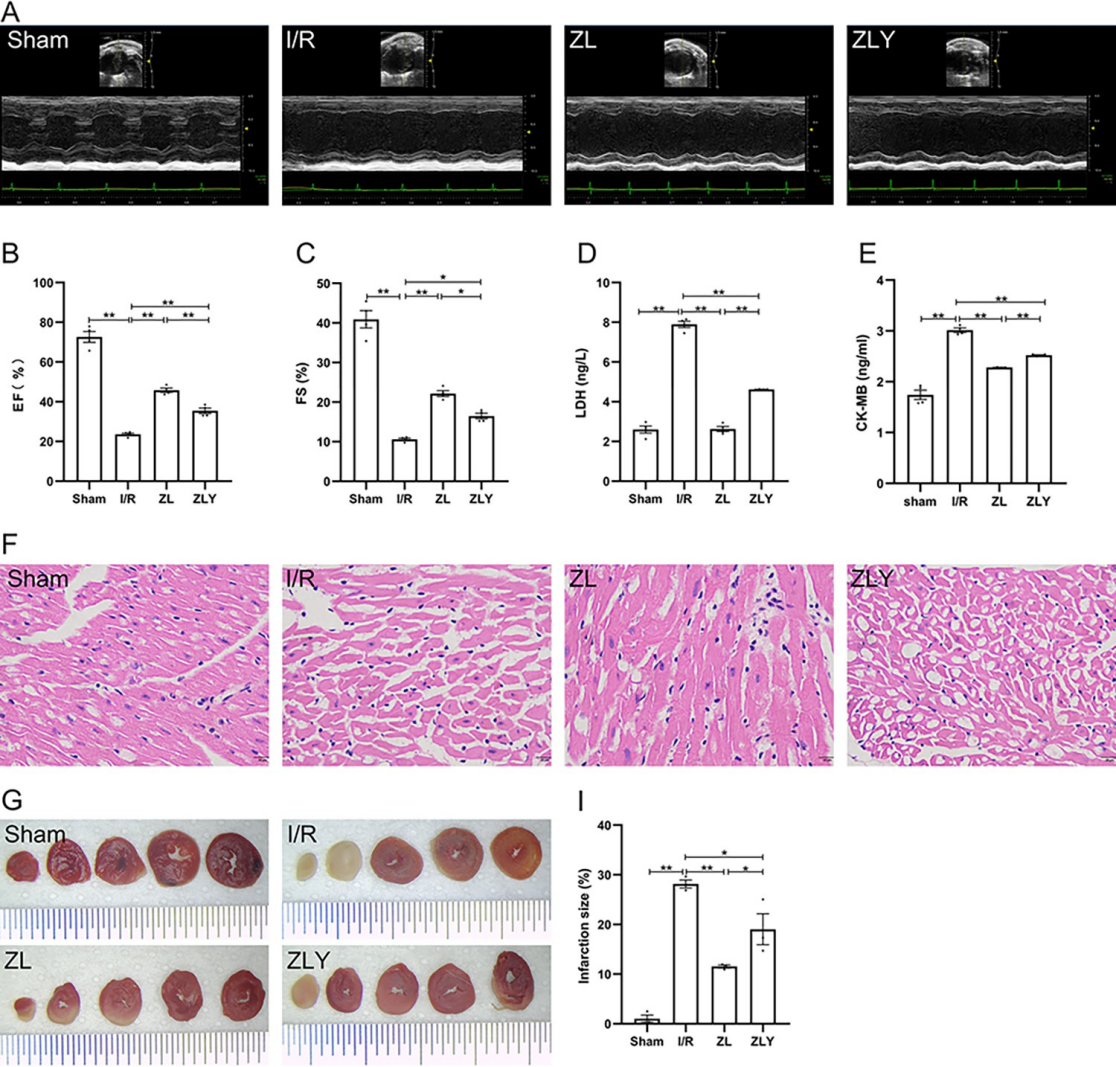
ZL protects mice from MI/RI. (A-C) Left ventricular ejection fraction (EF, %) and shortening fraction (FS, %) in mice were assessed by cardiac ultrasound (n = 4). (D, E) LDH and CK-MB activities were detected by enzyme-linked immunosorbent assay in serum (n = 4). (F) Histopathological pictures of heart tissue sections were stained with H&E. Scale bars represent 20 μm. (G, I) Representative images of infarct size were detected using TTC staining (n = 3). Data are shown as means ± S.E.D. **p < 0.01, *p < 0.05.

To assess the cardioprotective potential of ZL against MI/RI, we established an MI/RI mouse model. Cardiac ultrasound revealed a significant reduction in EF and FS values in the I/R group compared to the Sham group (p < 0.01, Fig 1A–1C). Notably, ZL pretreatment significantly ameliorated EF and FS values (p < 0.01). Furthermore, ELISA assays demonstrated increased LDH and CK-MB activities in the I/R group compared to the Sham group (p < 0.01, Fig 1D and 1E), whereas ZL pretreatment reversed these effects (p < 0.01). H&E staining revealed disrupted cell morphology, structural abnormalities, and myocardial fiber damage in the I/R group, which were notably reversed by ZL pretreatment (Fig 1F and in S1 File). Additionally, mice in the I/R group displayed obvious infarction compared to the ZL group, which showed significantly reduced cardiac infarction size (p < 0.05 or p < 0.01, Fig 1G and 1I)).

### ZL alleviated MI/RI by inhibiting ferroptosis

**Fig 2:**
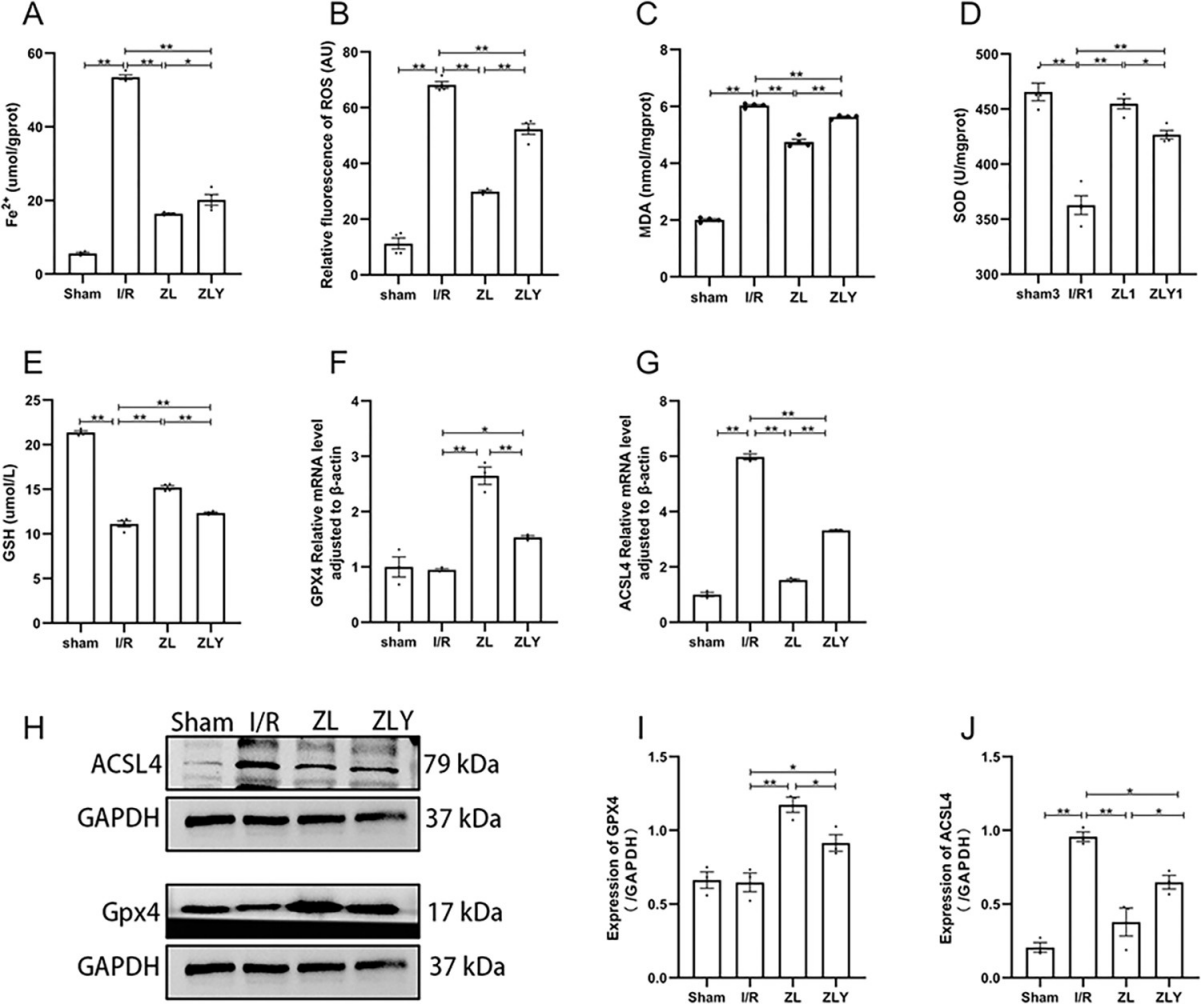
ZL-mediated alleviation of MI/RI by inhibiting ferroptosis. (A-E) The expression of Fe^2+^, ROS, MDA, SOD, and GSH were quantified (n = 4). (F-G) The expression of GPX4 and ACSL4 was detected by real-time quantitative polymerase chain reaction in cardiac tissue (n = 3). (H-J) The expression of GPX4 and ACSL4 was detected by western blot in cardiac tissue (n = 3). Data are shown as means ± S.E.D. **p < 0.01, *p < 0.05.

Compared to the Sham group, the I/R group exhibited significantly increased ROS, MDA, and Fe^2+^ levels, along with upregulated ACSL4 expression and downregulated SOD, GSH activity, and GPX4 expression (p < 0.05). ZL and ZLY groups showed significantly decreased ROS, MDA, and Fe^2+^ levels, downregulated ACSL4 expression, and upregulated SOD, GSH activity, and GPX4 expression compared to the I/R group (p < 0.05). Notably, the ZLY group displayed increased ROS, MDA, and Fe^2+^ levels, upregulated ACSL4 expression, and decreased SOD, GSH activity, and GPX4 expression compared to the ZL group, as shown in Fig 2 and in S2 File. (p < 0.05).

### ZL promotes the expression and activation of Nrf2 and the HO-1/GPX4 axis during MI/RI

**Fig 3:**
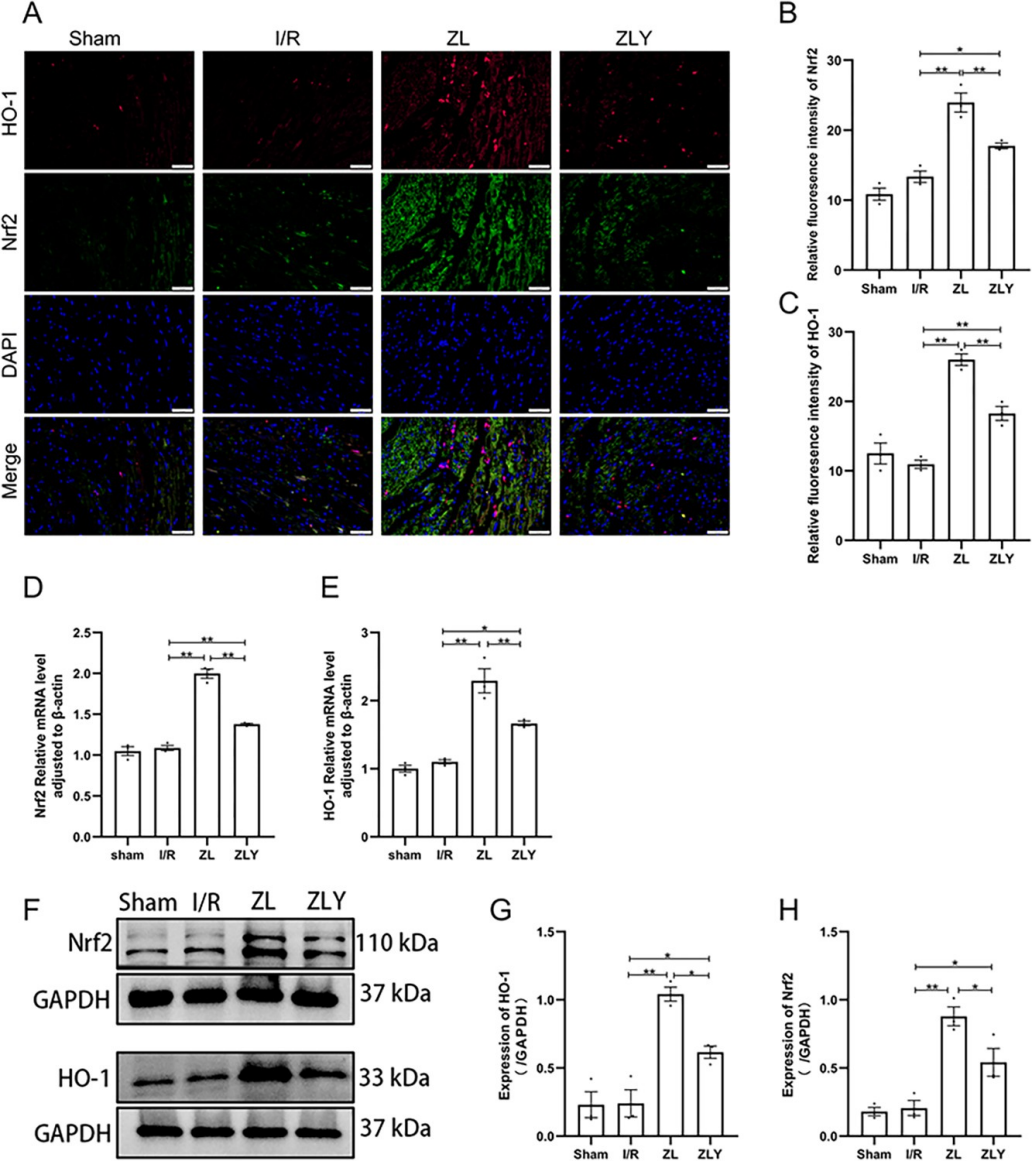
ZL promotion of Nrf2 expression and activation of the HO-1/GPX4 axis during MI/RI. (A-C) Immunofluorescent staining visualized the expression of Nrf2 (green), HO-1 (red), and nuclei (blue) in myocardial tissues. Scale bars: 20 μm. Images captured at × 400 magnification. (D, E) RT-qPCR assessed the expression of Nrf2 and HO-1 in cardiac tissue. (F-H) The expression of Nrf2 and HO-1 was detected by western blot in cardiac tissue. Data are shown as means ± S.E.D (n = 3). **p < 0.01, *p < 0.05.

To investigate the relationship between ZL’s ferroptosis-inhibiting effect and the Nrf2/HO-1 axis, we examined the mRNA and protein levels of Nrf2 and HO-1. Compared to the I/R group, the ZL and ZLY groups showed significantly upregulated Nrf2 and HO-1 mRNA and protein levels (p < 0.05). Furthermore, Nrf2 and HO-1 mRNA and protein levels were downregulated in the ZLY group compared to the ZL group (p < 0.05, Fig 3D–3H and in S2 File). Immunofluorescence results confirmed the changes observed in RT-qPCR and Western blot analyses (p < 0.05 or p < 0.01, Fig 3A–3C and in S1 File).

### ZL-promoted Nrf2 upregulation was mediated by the PI3K/AKT pathway

**Fig 4:**
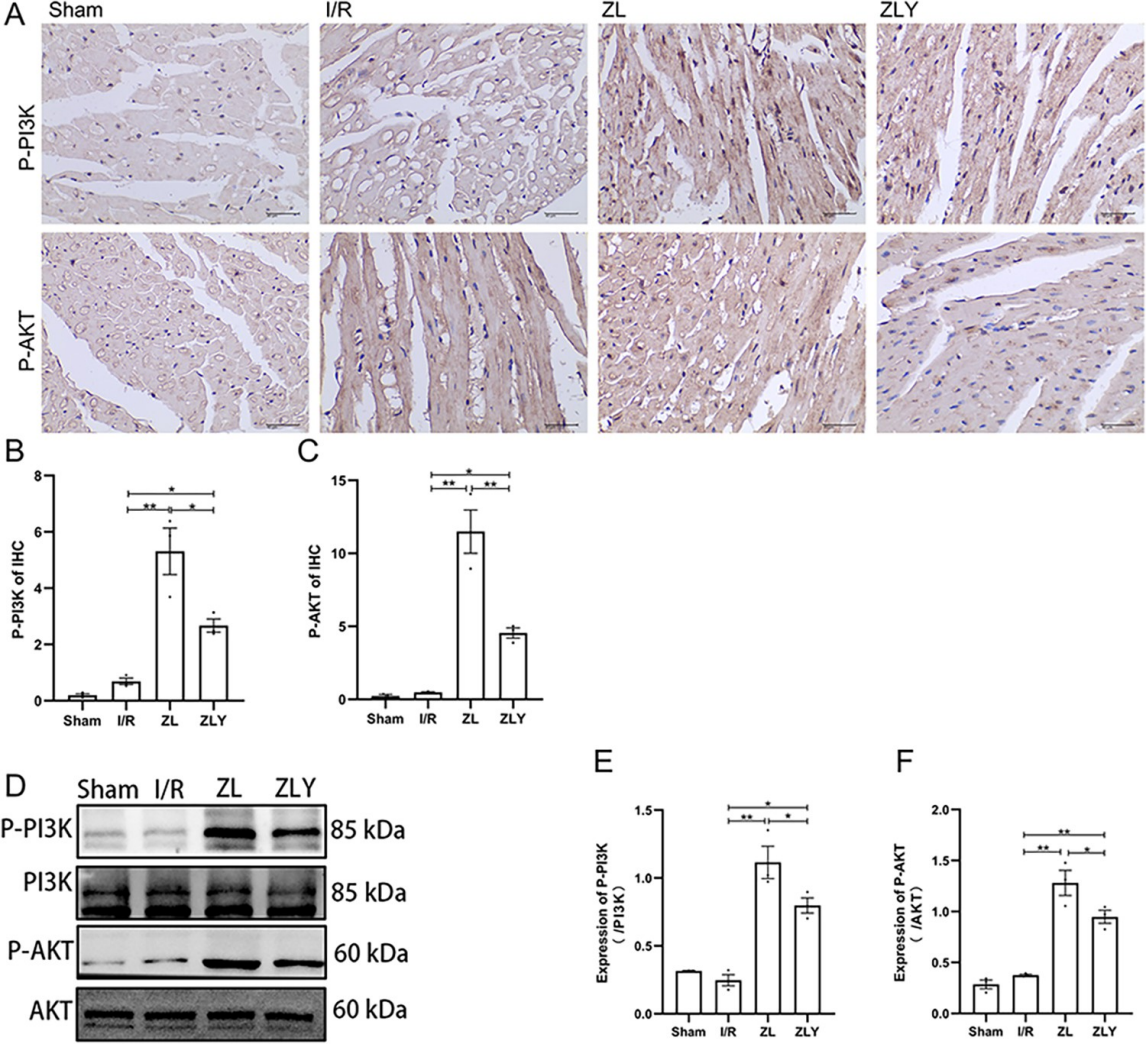
ZL-promoted Nrf2 upregulation was mediated by the PI3K/AKT pathway. (A-C) The expression of p-PI3K and p-AKT in myocardial tissue was detected by immunohistochemistry. Scale bars represent 20 μm. Images were captured at × 400 magnification. (D-F) The expression of p-PI3K and p-AKT was detected by western blot in cardiac tissue. Data are shown as means ± S.E.D (n = 3). **p < 0.01, *p < 0.05.

Compared to the Sham group, the expression of p-PI3K and p-AKT in the I/R group did not show significant alterations (p > 0.05). Conversely, the ZL and ZLY groups displayed significantly upregulated p-PI3K and p-AKT expression compared to the I/R group (p < 0.05). Furthermore, the ZLY group exhibited downregulated p-PI3K and p-AKT expression compared to the ZL group (p < 0.05, Fig 4D-4F and in S2 File). Immunohistochemistry results mirrored the changes observed in RT-qPCR and Western blot analyses (Fig 4A-4C and in S1 File).

## Discussion

Acute myocardial infarction is a major cause of death in the aging population, and reperfusion, while the most effective treatment, can worsen tissue damage [1, 15]. Therefore, preventing and mitigating MI/RI injury is crucial. Established markers like LDH and CK-MB can be harnessed to identify myocardial cell injury [16], while infarct size is the gold standard endpoint in both MI/RI and cardioprotection studies [17]. In this study, we investigated the protective effects of ZL against MI/RI in mice. Our findings demonstrate that ZL pretreatment significantly reduced serum LDH and CK-MB activity, improved cardiac function, lessened myocardial infarct area, and ameliorated pathological injuries, suggesting its potential for cardioprotection after MI/RI.

Ferroptosis, a recently discovered form of iron-dependent cell death linked to lipid peroxidation, has gained significant attention in the context of ischemia/reperfusion injury [18–20]. We provide direct evidence that ZL protects mouse hearts from MI/ RI. To investigate the potential role of ferroptosis in MI/RI, we measured ferroptosis markers like intracellular ferrous iron, lipid peroxidation products (MDA), ROS, GPX4, GSH, and ACSL4 [21, 22]. Myocardial levels of ferrous iron, ROS, MDA, and ACSL4 were significantly elevated, while GSH and GPX4 activity decreased after MI/RI, suggesting the induction of oxidative stress and ferroptosis. Notably, ZL preconditioning reversed these effects, mitigating myocardial damage from ferroptosis. These findings suggest that ZL’s cardioprotective effects are linked to its anti-ferroptosis and anti-oxidative stress properties.

Nrf2, a key regulator of intracellular redox homeostasis, plays a role in controlling lipid peroxidation [23]. Under oxidative stress, Nrf2 rapidly dissociates from Kelch-like epichlorohydrin-associated protein-1 (Keap1) and translocates from the cytoplasm to the nucleus, thereby triggering a series of gene transcription events. This process regulates cell survival, mitigates oxidative damage, and exerts a cellular protective effect [7]. Notably, HO-1, ACSL4, and GPX4, enzymes and proteins crucial for preventing lipid peroxidation, are Nrf2 target genes and are also implicated in ferroptosis [24, 25]. To investigate the mechanism, we designed experiments to determine whether ZL could activate Nrf2 and modulate the expression of HO-1 and GPX4 (anti-ferroptotic) while inhibiting ACSL4 (pro-ferroptotic). Our results revealed that ZL pretreatment significantly increased Nrf2 expression compared to the I/R group. Furthermore, ZL pretreatment significantly upregulated HO-1 and GPX4 expression while downregulating ACSL4 levels compared to the I/R group. These findings collectively suggest that ZL’s cardioprotective effects are mediated by activating Nrf2 to promote HO-1/GPX4 expression and suppress ferroptosis.

The PI3K/AKT signaling pathway regulates Nrf2 expression and activity [26]. This pathway is crucial for cell survival, growth, and death regulation [27–29]. It also plays a role in ferroptosis, with its inhibition promoting ferroptosis [30, 31]. Studies suggest PI3K/AKT activation upregulates Nrf2, leading to HO-1 expression and protection against I/R injury in cardiomyocytes [32]. Consistent with these findings, ZL pretreatment increased p-PI3K, p-AKT, and Nrf2 protein levels in myocardial tissues. Conversely, the ZLY group treated with PI3K/AKT pathway inhibitors displayed decreased p-PI3K, p-AKT, and Nrf2 protein expression compared to the ZL group.

## Conclusion

The present study demonstrates the cardioprotective effects of ZL from MI/RI mediated by inhibiting ferroptosis through activation of the PI3K/AKT/Nrf2 signaling pathway. These findings provide compelling evidence supporting the potential of ZL as a therapeutic strategy to combat ferroptosis induced by MI/RI, warranting further investigation in clinical settings.

## Supporting information

S1 File S1_raw_images. (PDF)

S2 File Uncropped and unadjusted images underlying all blot or gel results. (PDF)

S1 Table Material and reagents. (DOCX)

S2 Table Primers used for RT-PCR. (DOCX)
