# Supplementary material for: Zhilong Huoxue Tongyu capsule improves myocardial ischemia/reperfusion injury via the PI3K/AKT/Nrf2 axis
Source: PLoS One. 2024 Apr 30;19(4):e0302650. doi: 10.1371/journal.pone.0302650 (PMC11060539; doi:10.1371/journal.pone.0302650)
Supplement: S1 File — (PDF) [file pone.0302650.s001.pdf]

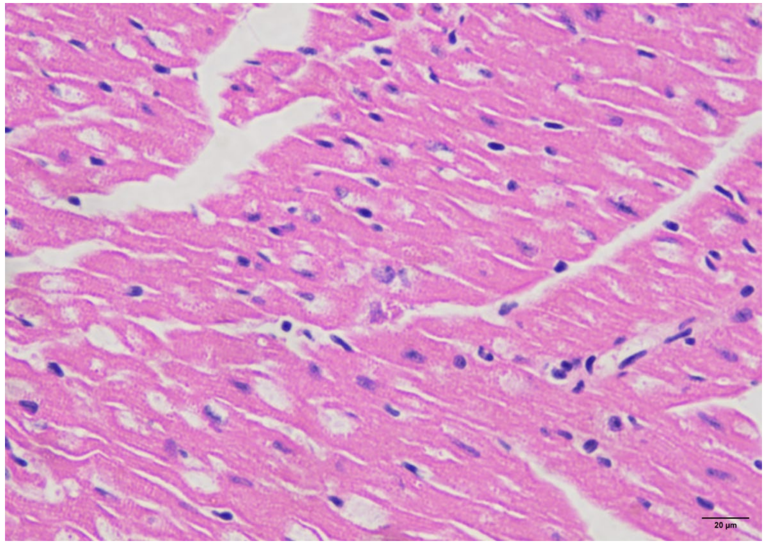

Original Image for Fig 1 (Sham)

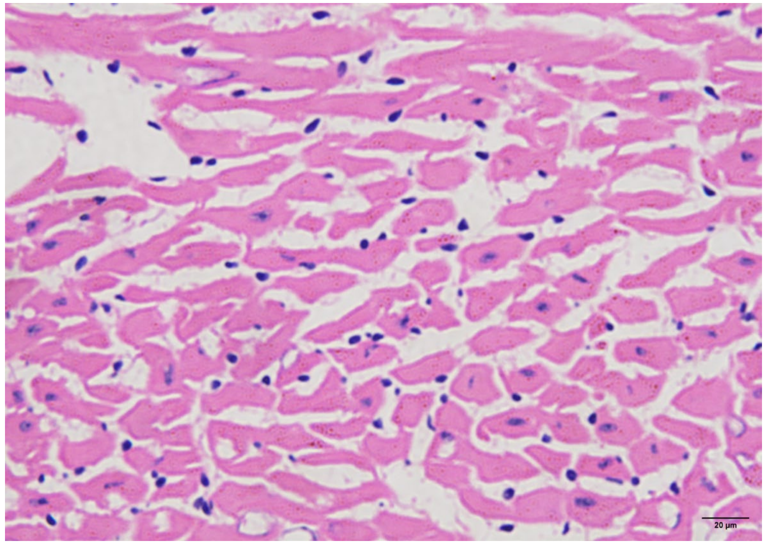

Original Image for Fig 1 (I/R)

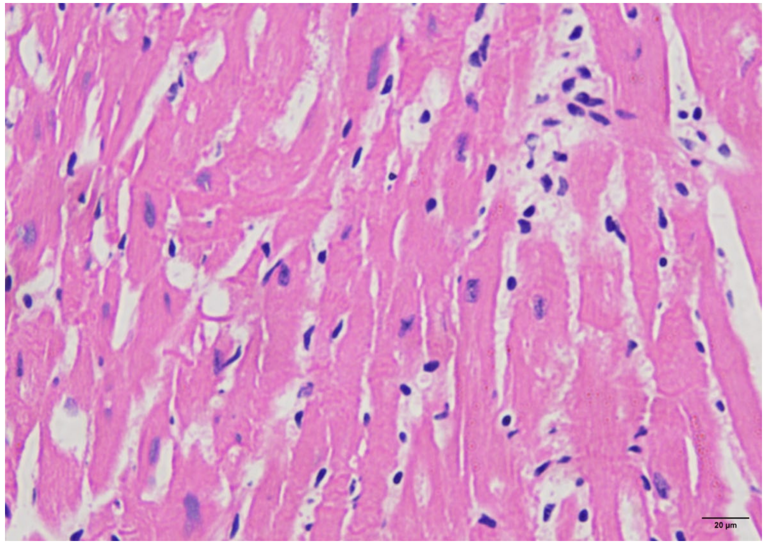

Original Image for Fig 1 (ZL)

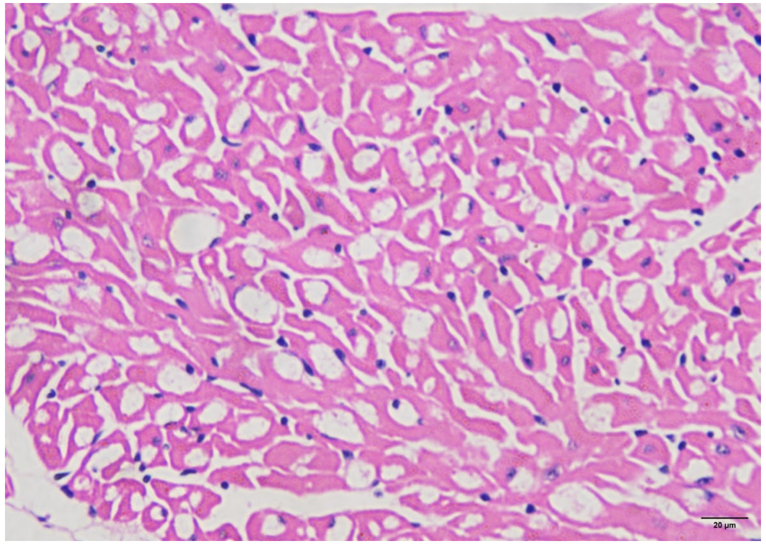

Original Image for Fig 1 (ZLY)

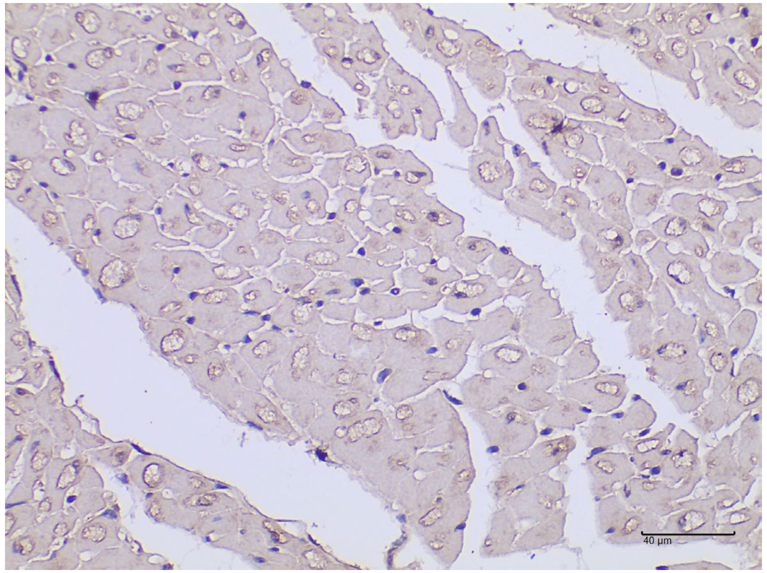

Original Image for Fig 4 (p-PI3K, Sham)

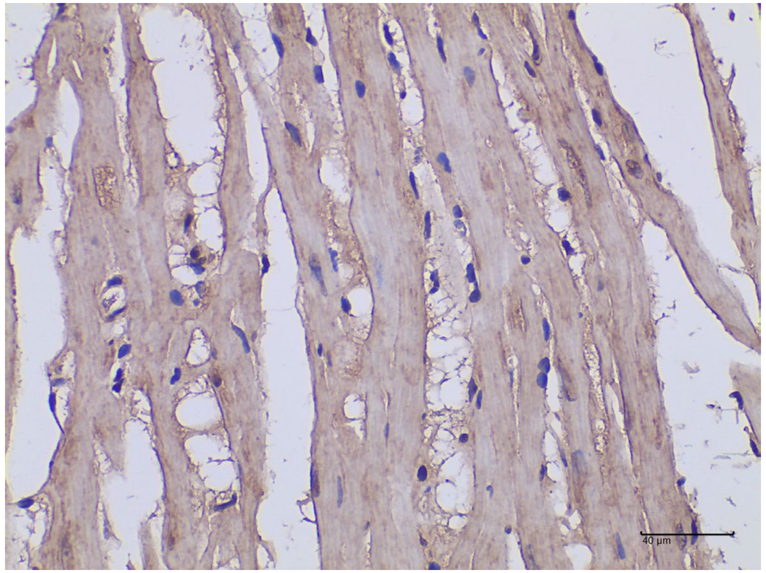

Original Image for Fig 4 (p-PI3K, I/R)

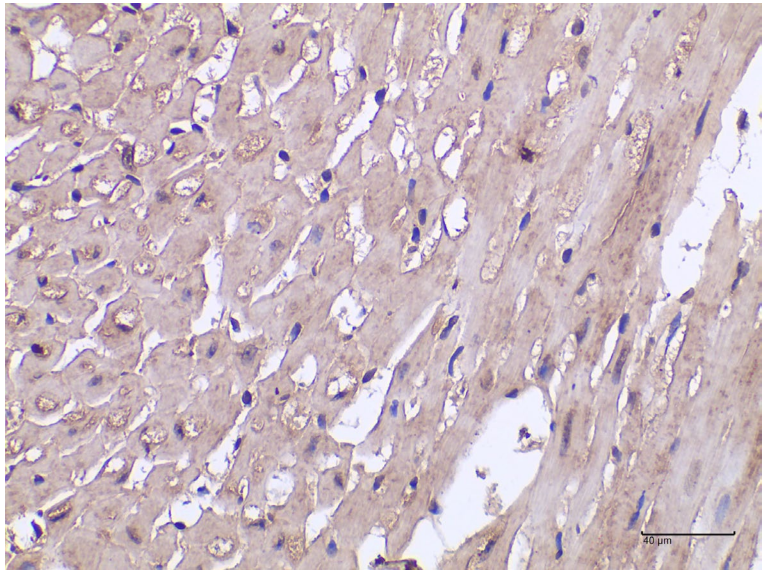

Original Image for Fig 4 (p-PI3K, ZL)

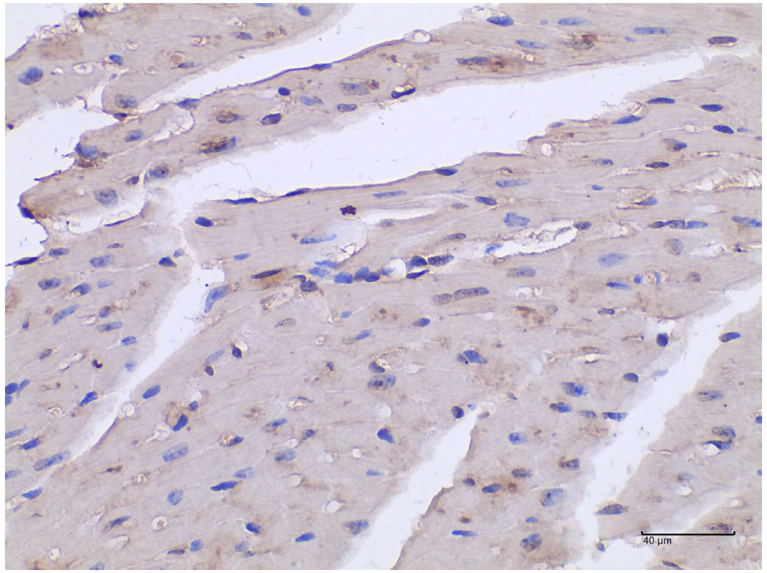

Original Image for Fig 4 (p-PI3K, ZLY)

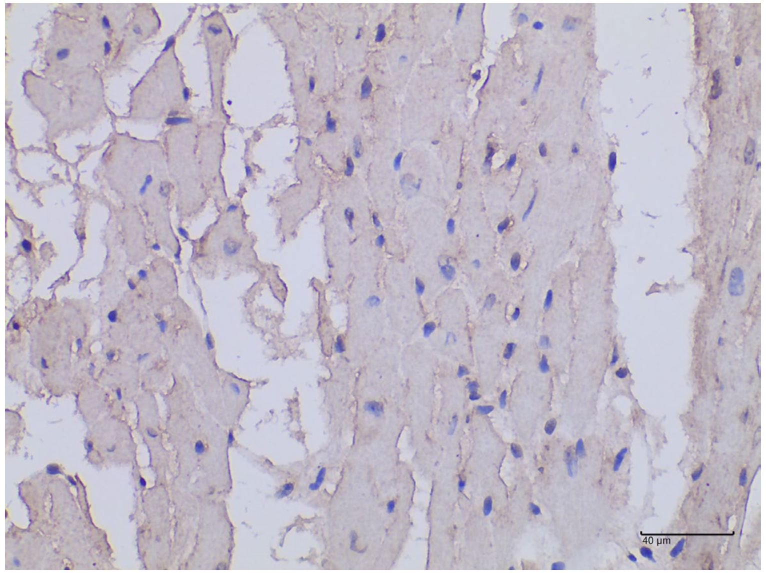

Original Image for Fig 4 (p-AKT, Sham)

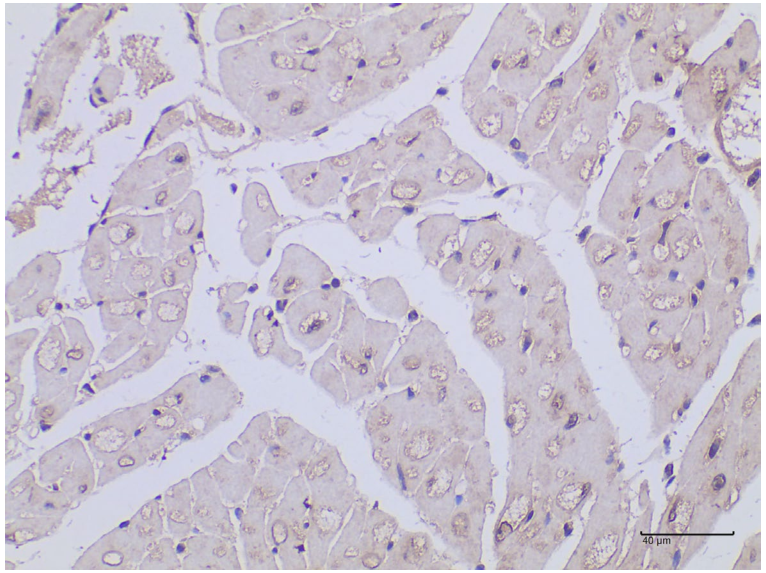

Original Image for Fig 4 (p-AKT, I/R)

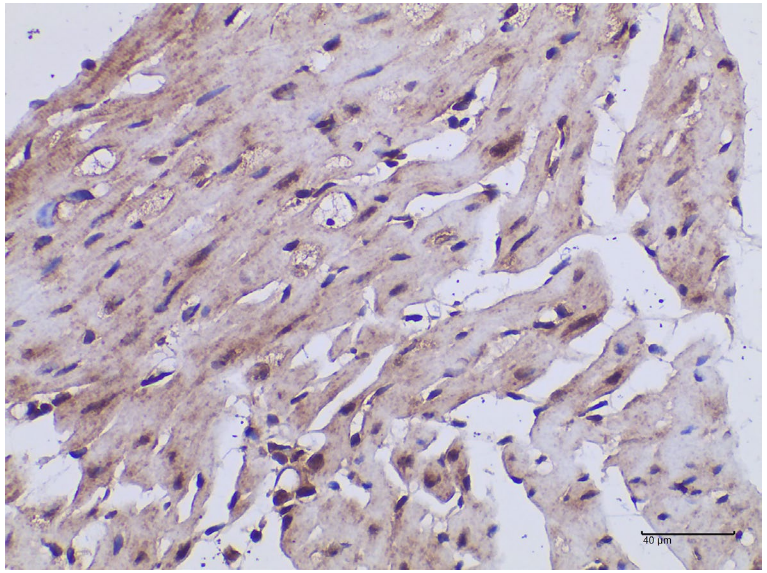

Original Image for Fig 4 (p-AKT , ZL)

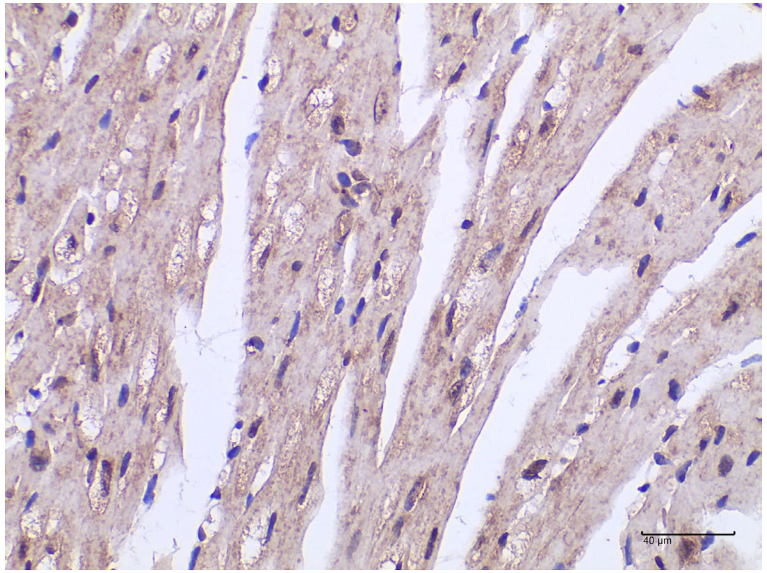

Original Image for Fig 4 (p-AKT, ZLY)

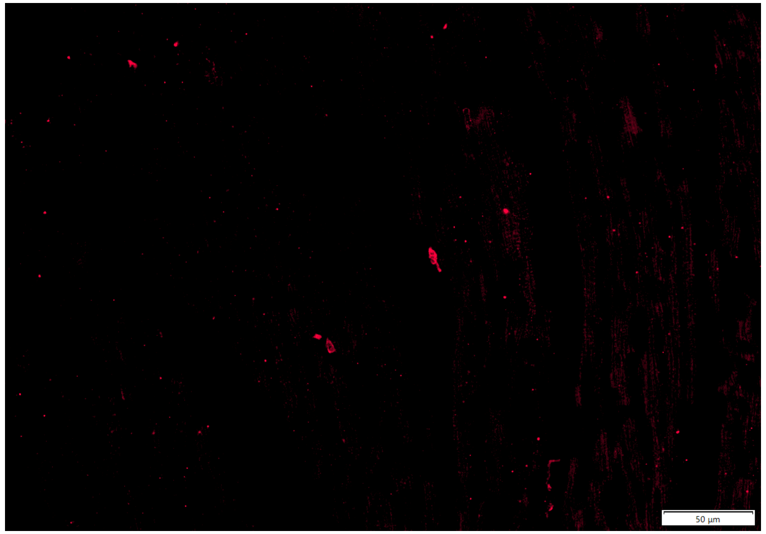

Original Image for Fig 3 (HO-1, Sham)

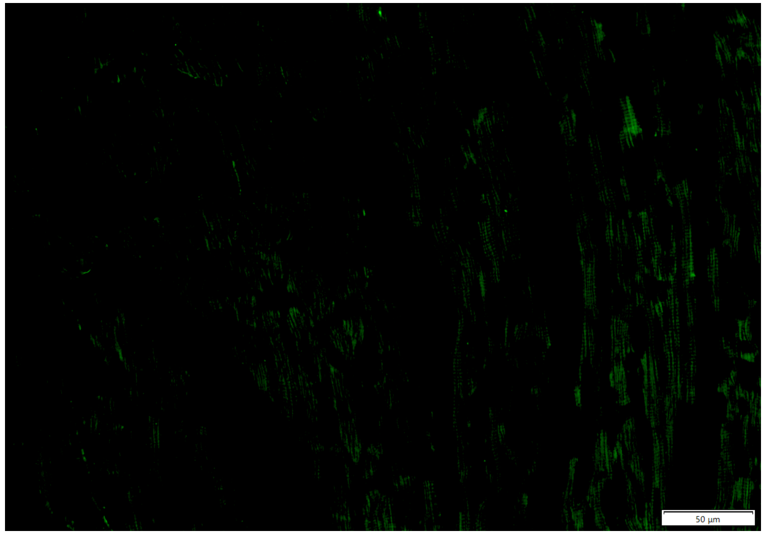

Original Image for Fig 3 (Nrf2, Sham)

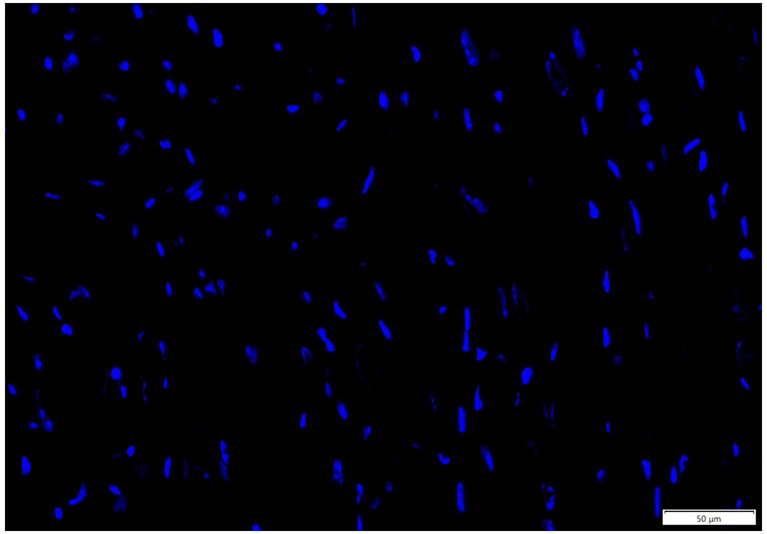

Original Image for Fig 3 (DAPI, Sham)

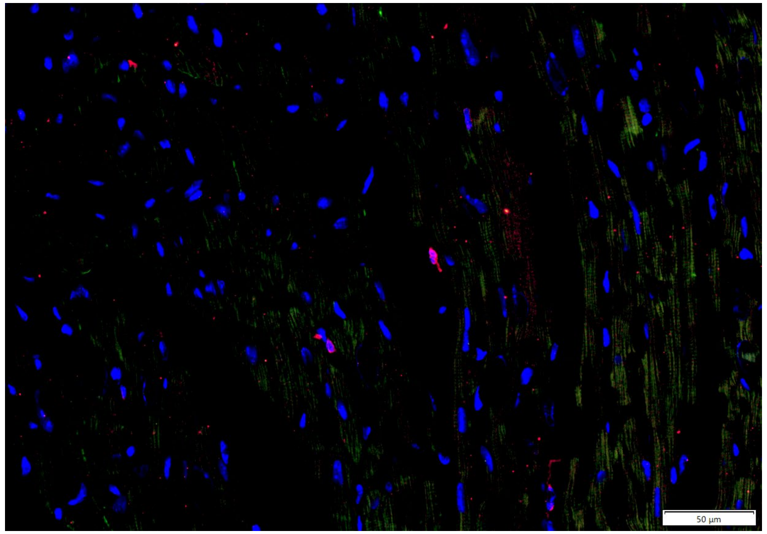

Original Image for Fig 3 (Merge, Sham)

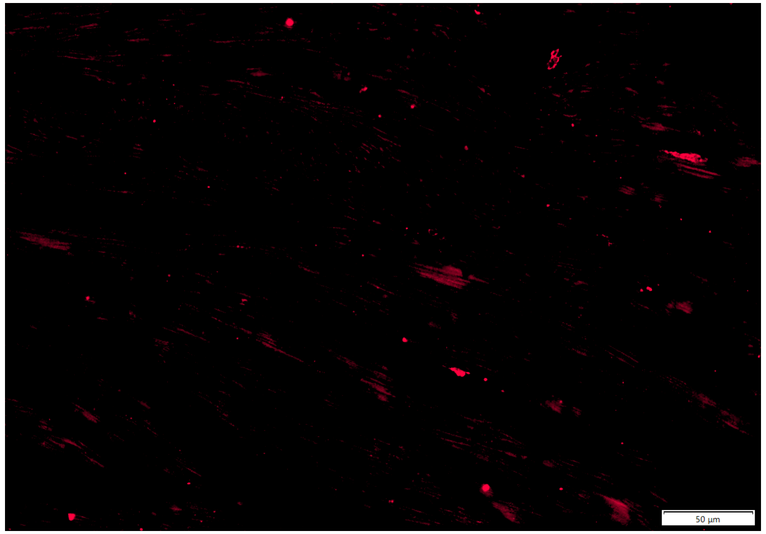

Original Image for Fig 3 (HO-1, I/R)

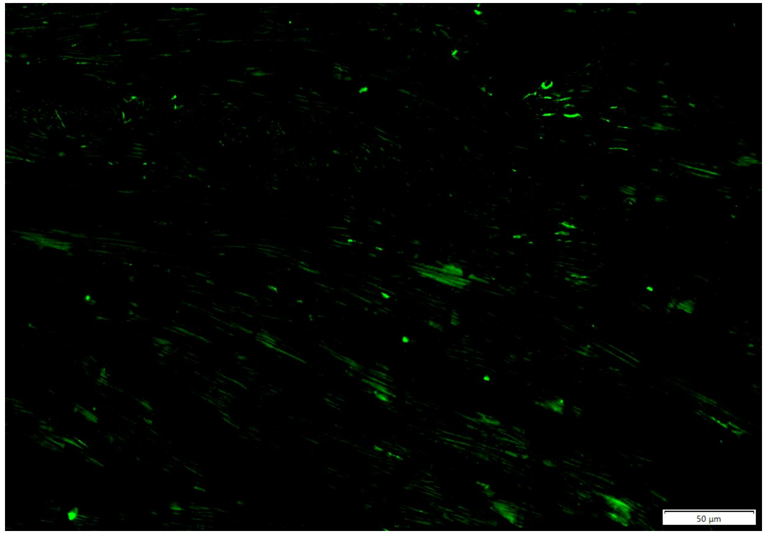

Original Image for Fig 3 (Nrf2, I/R)

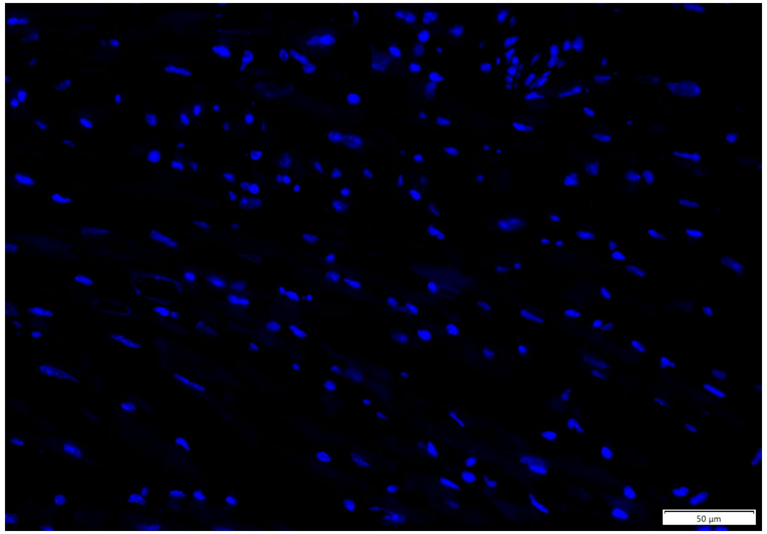

Original Image for Fig 3 (DAPI, I/R)

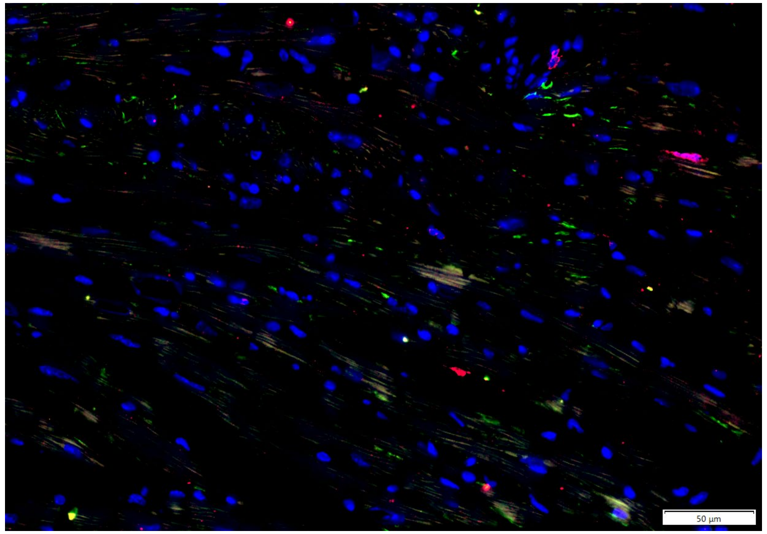

Original Image for Fig 3 (Merge, I/R)

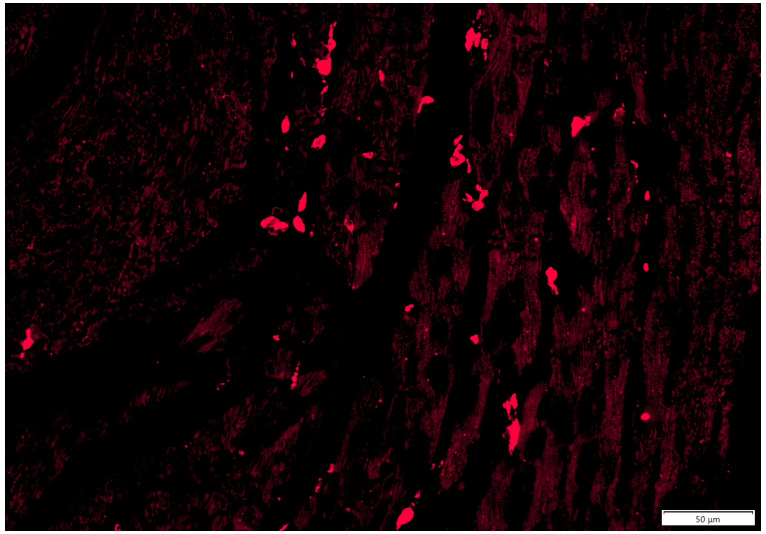

Original Image for Fig 3 (HO-1, ZL)

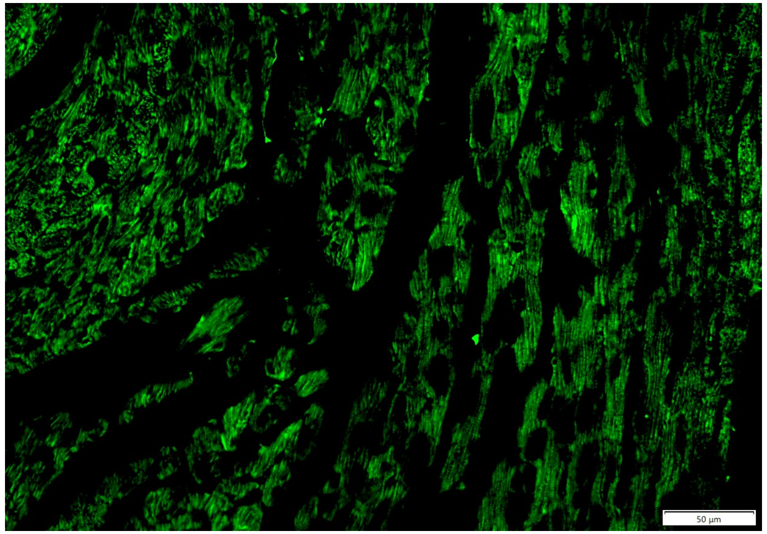

Original Image for Fig 3 (Nrf2, ZL)

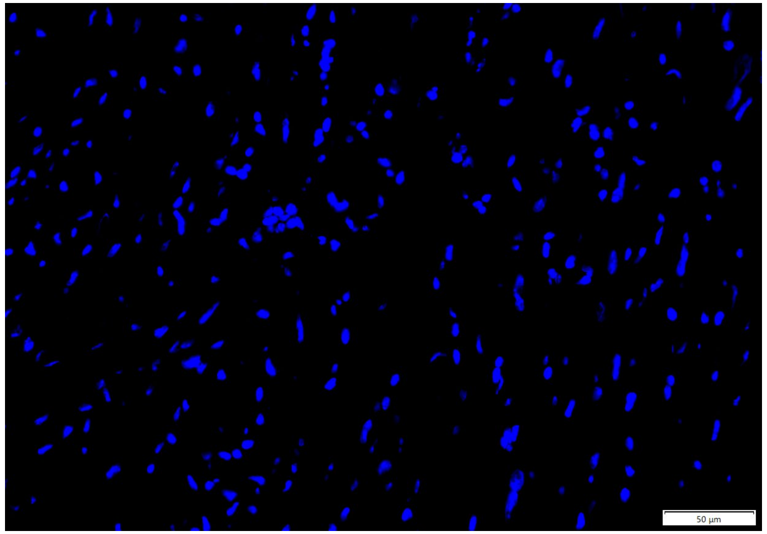

Original Image for Fig 3 (DPAI, ZL)

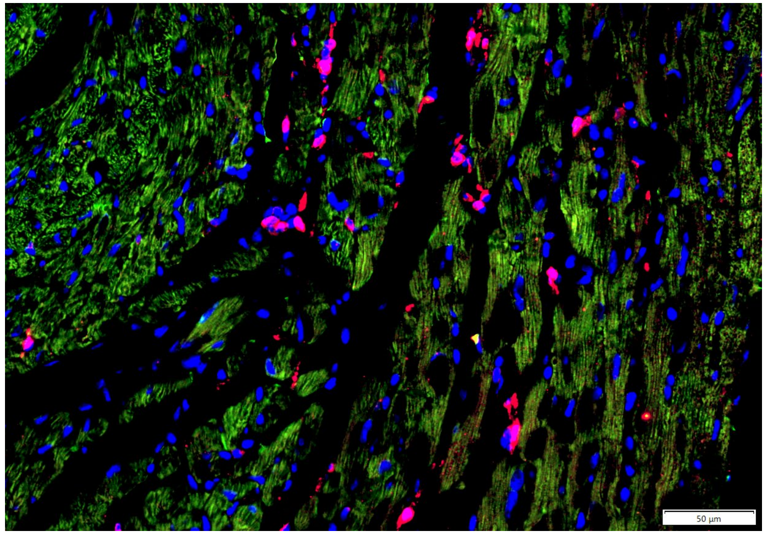

Original Image for Fig 3 (Merge, ZL)

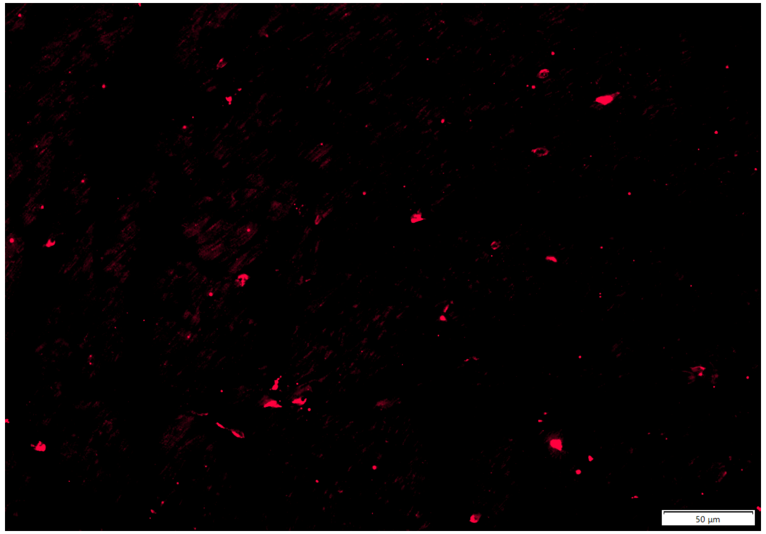

Original Image for Fig 3 (HO-1, ZLY)

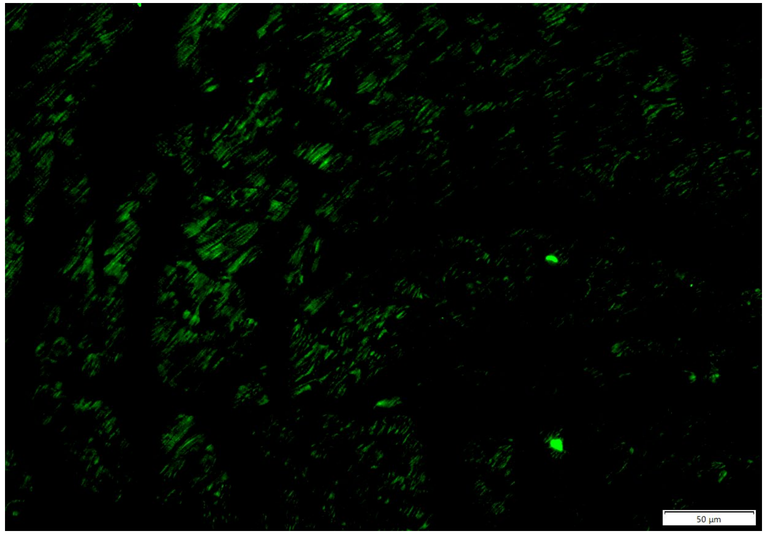

Original Image for Fig 3 (Nrf2, ZLY)

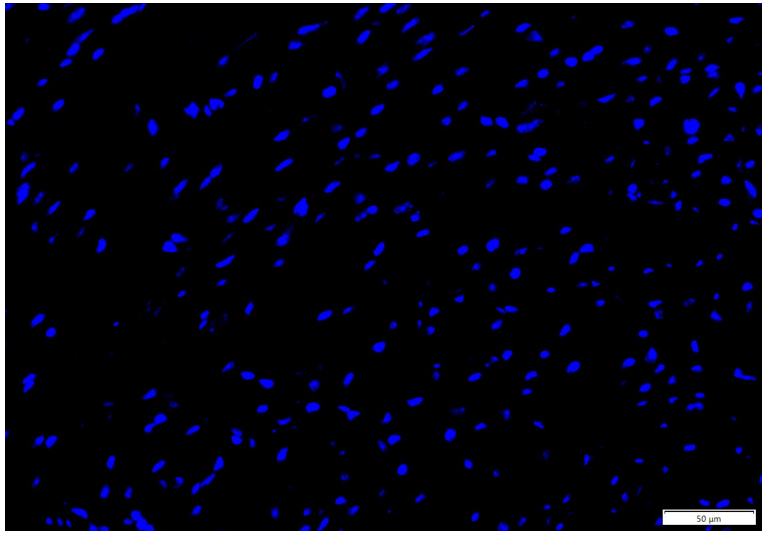

Original Image for Fig 3 (DPAI, ZLY)

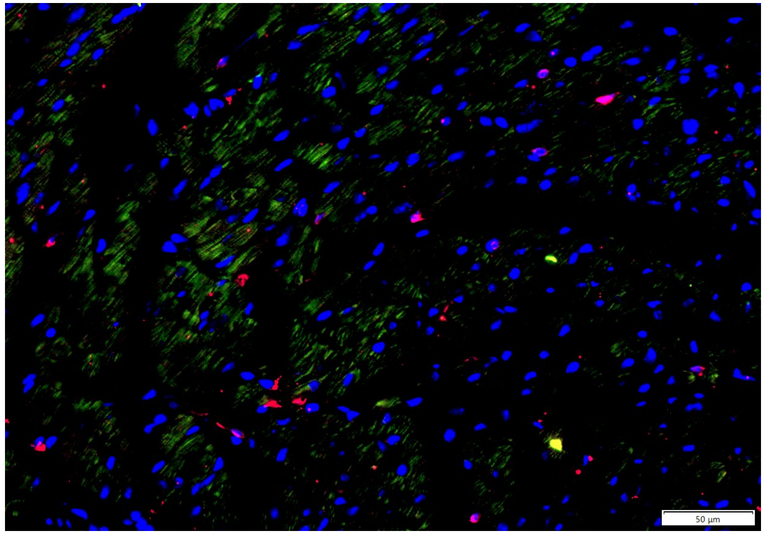

Original Image for Fig 3 (Merge, ZLY)

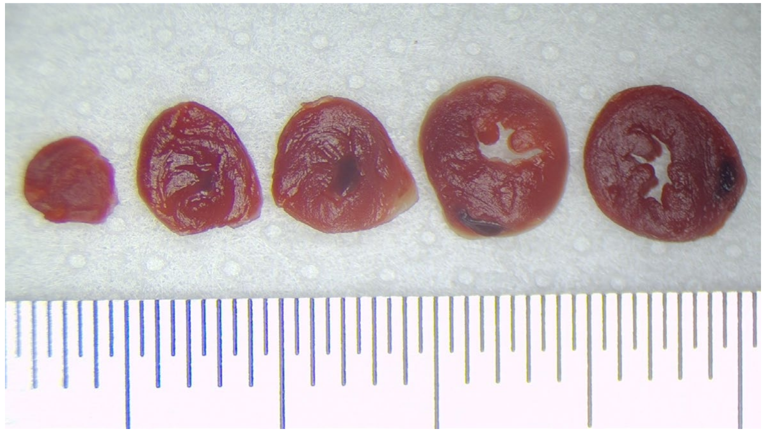

Original Image for Fig 1 (Sham)

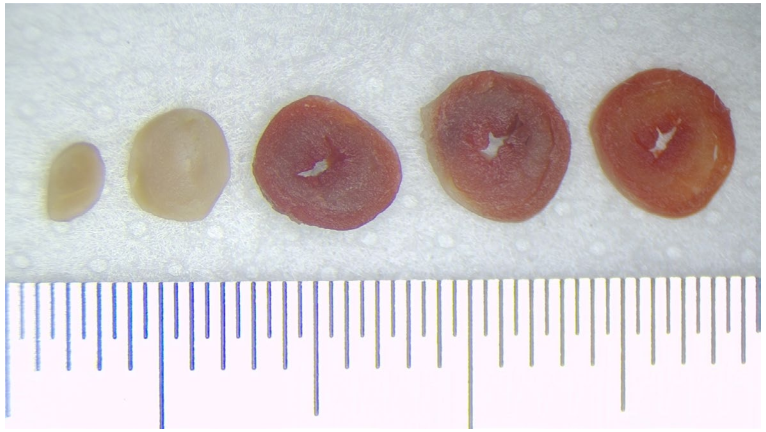

Original Image for Fig 1 (I/R)

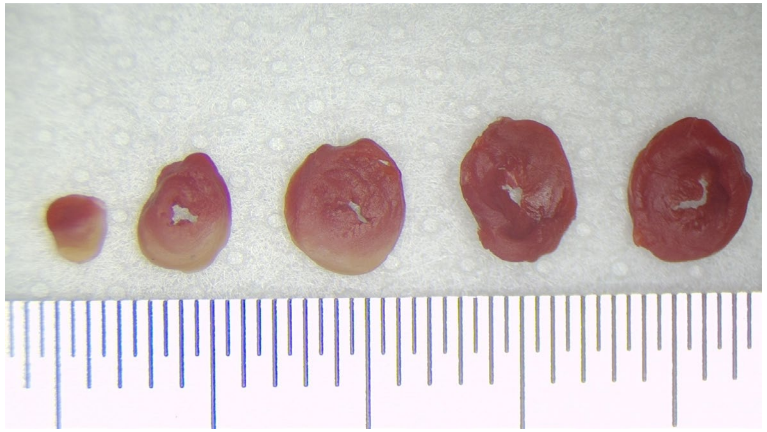

Original Image for Fig 1 (ZL)

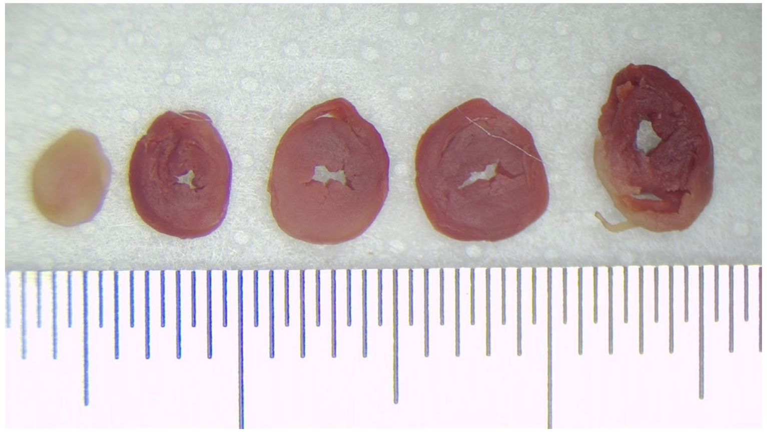

Original Image for Fig 1 (ZLY)
