# Supplementary material for: Zhilong Huoxue Tongyu capsule improves myocardial ischemia/reperfusion injury via the PI3K/AKT/Nrf2 axis
Source: PLoS One. 2024 Apr 30;19(4):e0302650. doi: 10.1371/journal.pone.0302650 (PMC11060539; doi:10.1371/journal.pone.0302650)
Supplement: S2 File — (PDF) [file pone.0302650.s002.pdf]

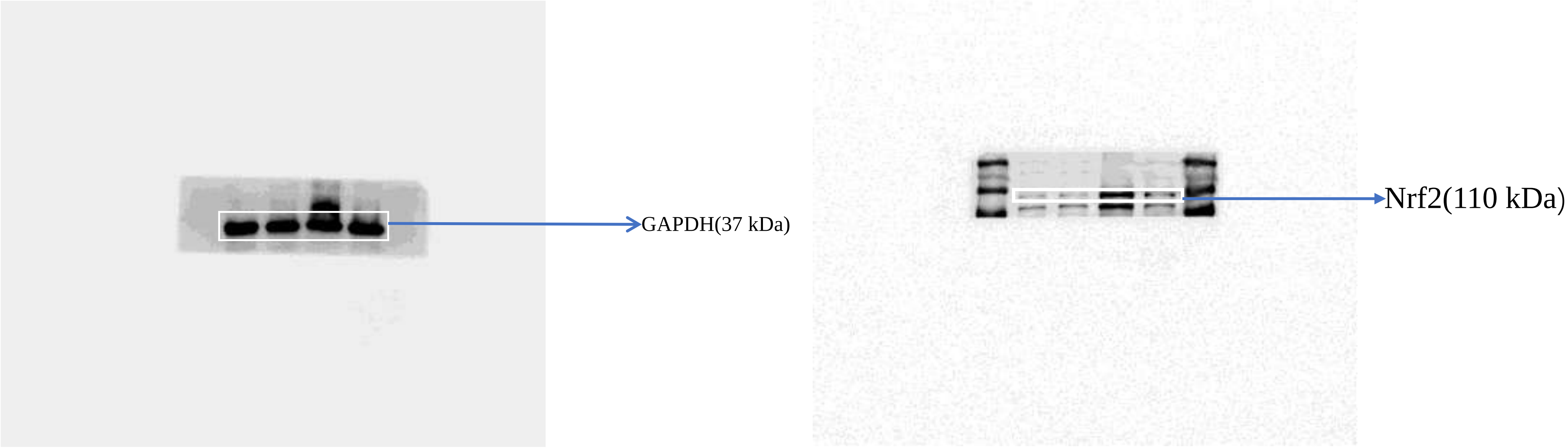

The labeled places represent Nrf2 in the western blot analysis of Figure 3

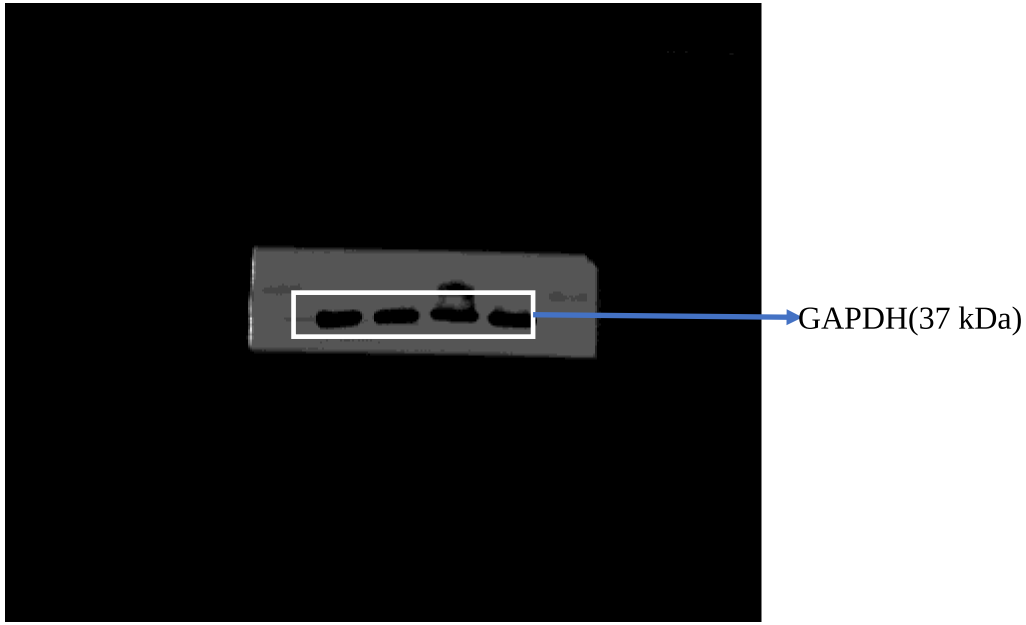
GAPDH(37 kDa)

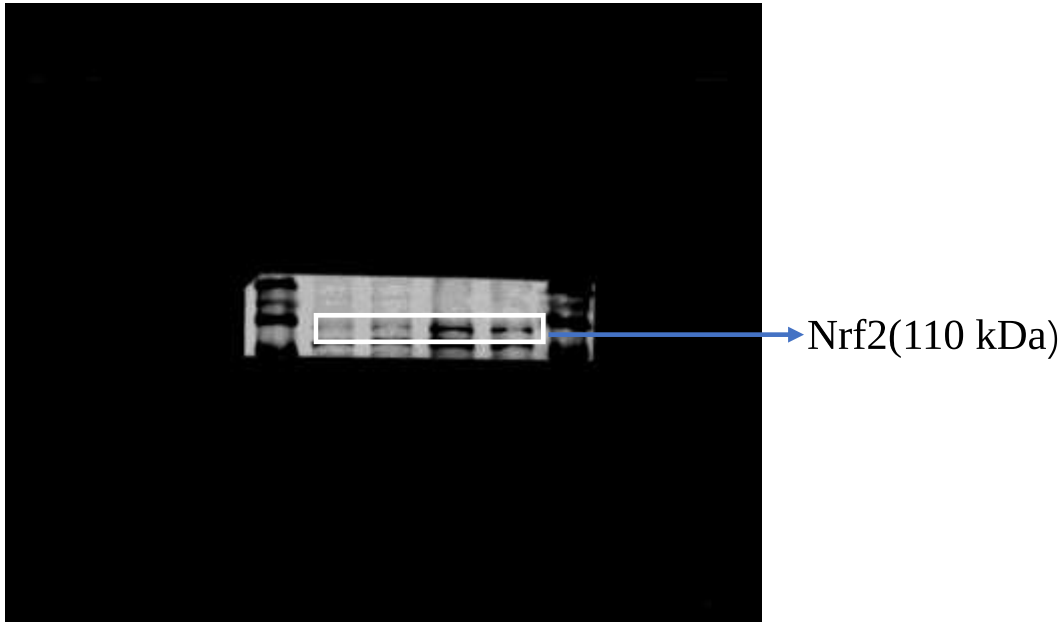
Nrf2(110 kDa)

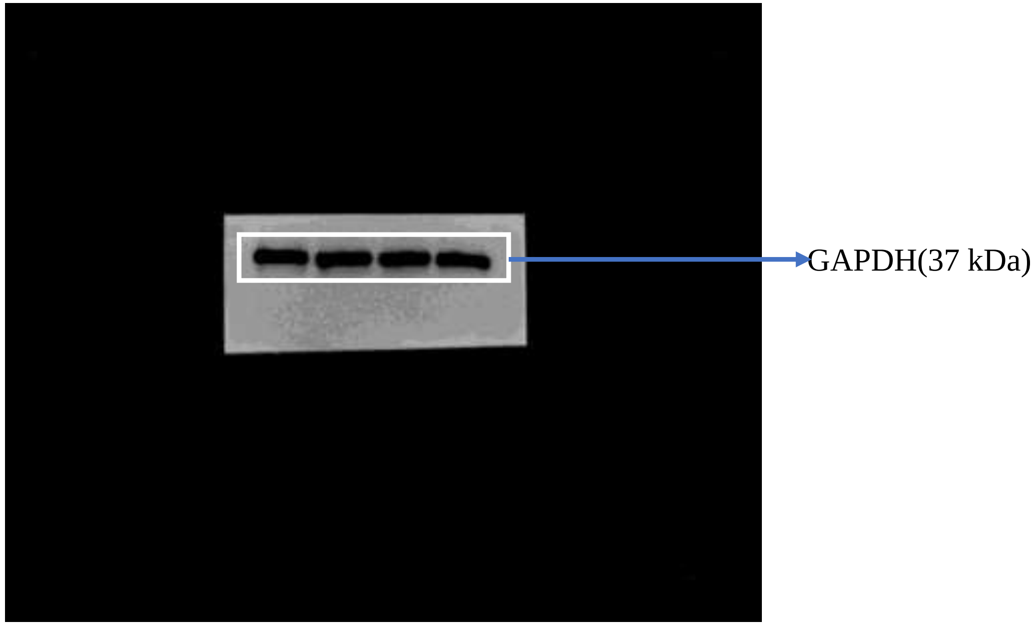
GAPDH(37 kDa)

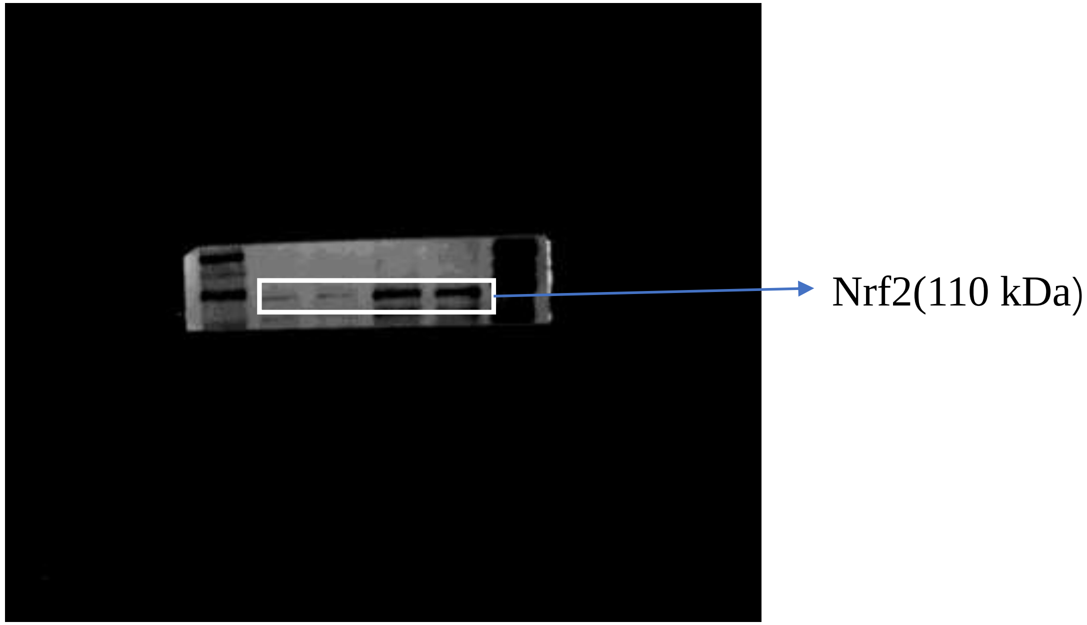
Nrf2(110 kDa)

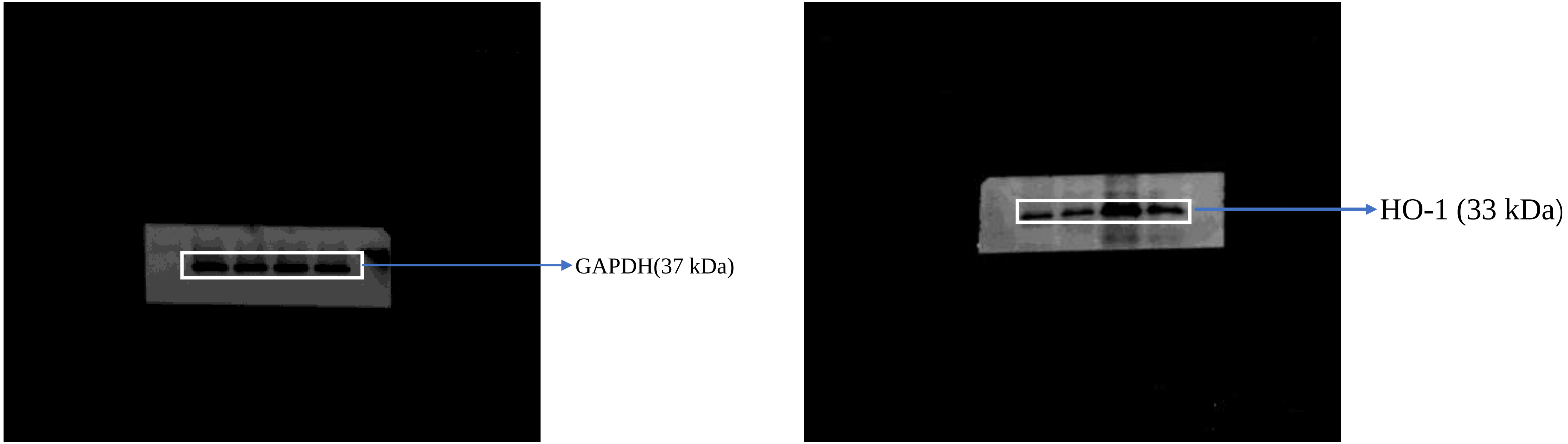

The labeled places represent HO-1 in the western blot analysis of Figure 3

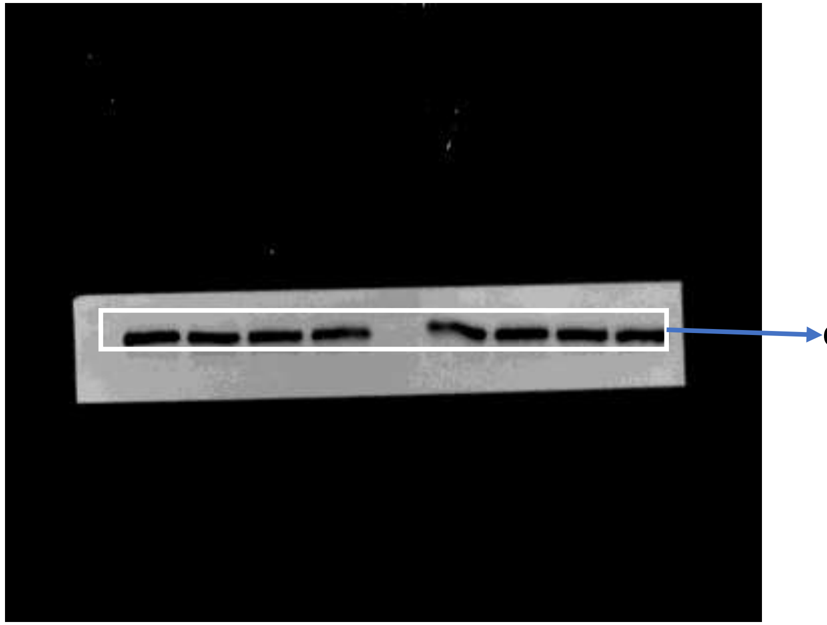

GAPDH(37 kDa)

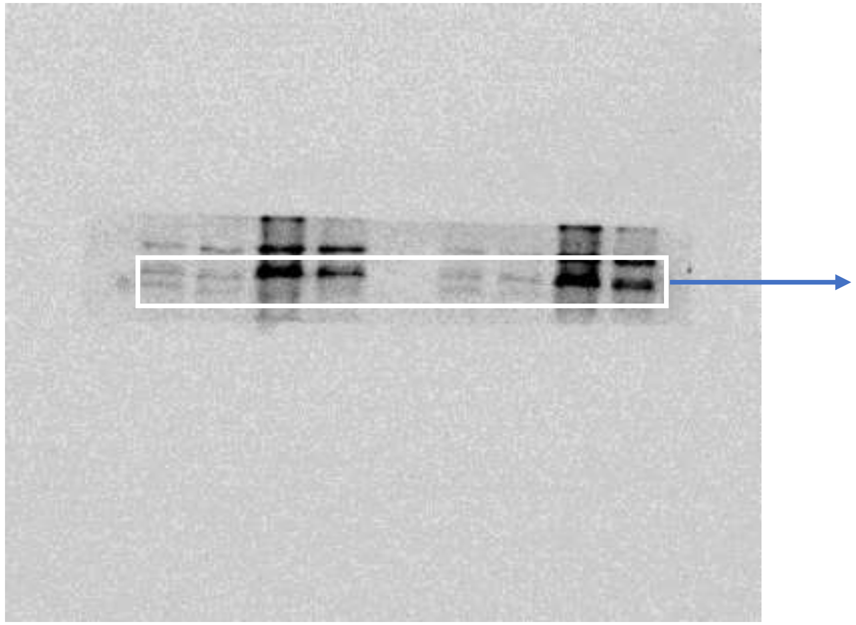

HO-1 (33 kDa)

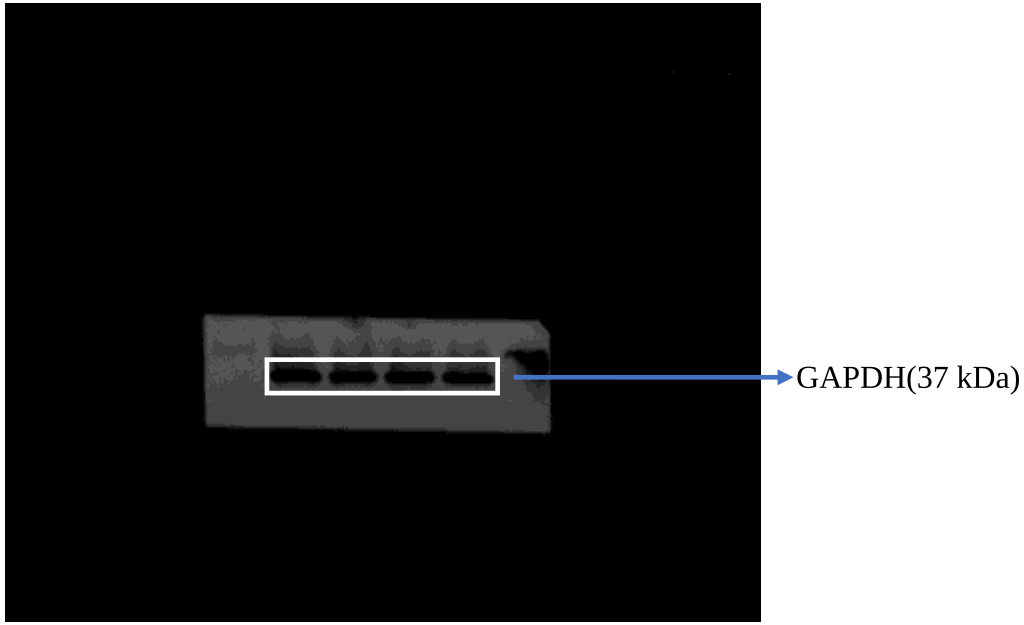
GAPDH(37 kDa)

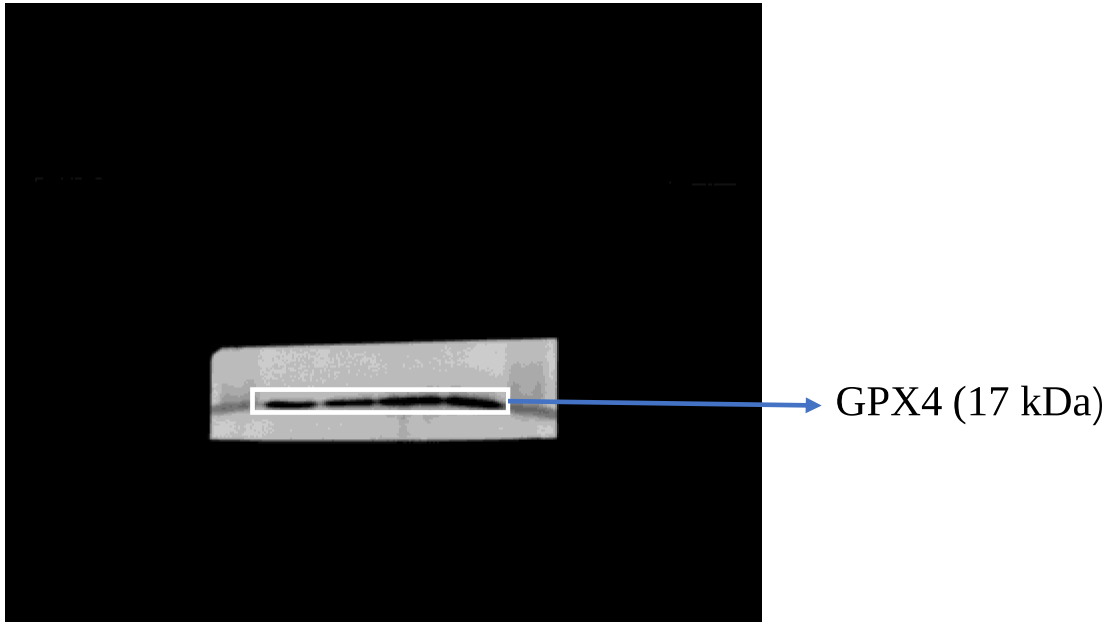
GPX4 (17 kDa)

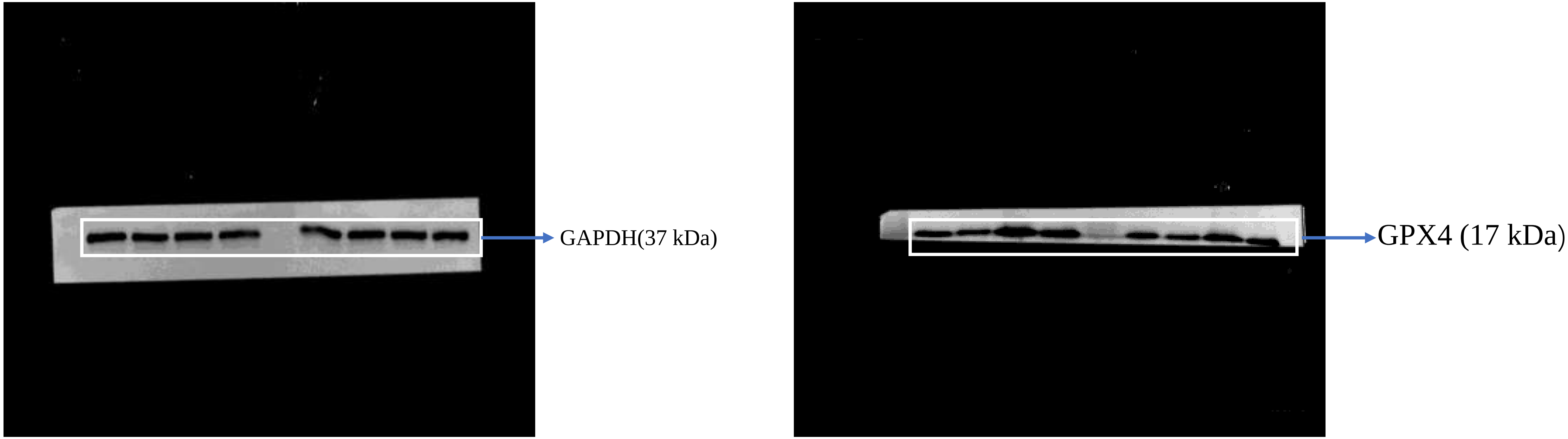

The labeled places represent GPX4 in the western blot analysis of Figure 2

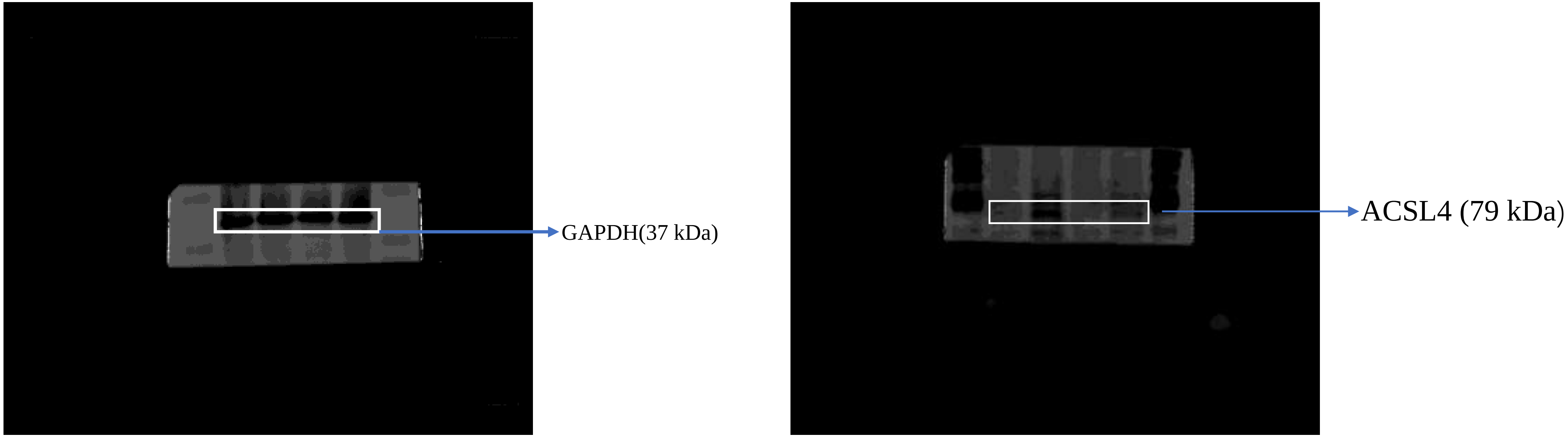
GAPDH(37 kDa)
ACSL4 (79 kDa)

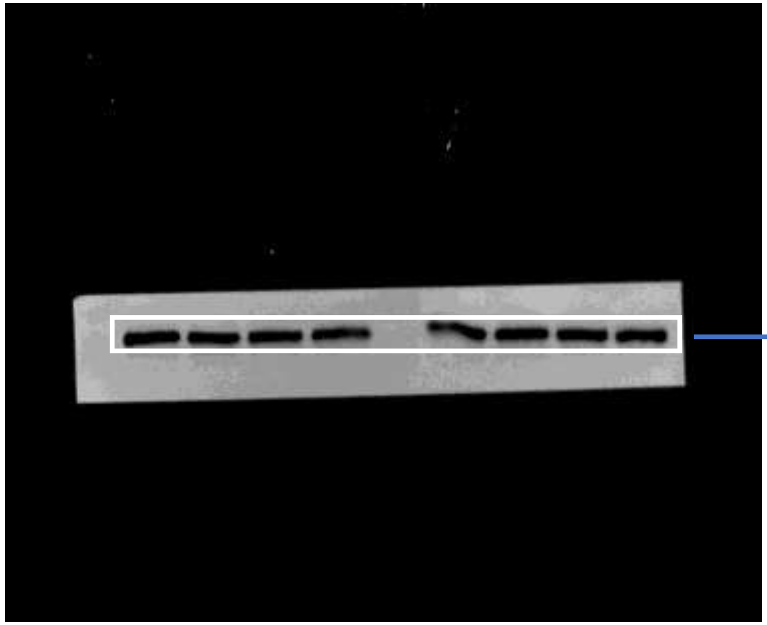

GAPDH(37 kDa)

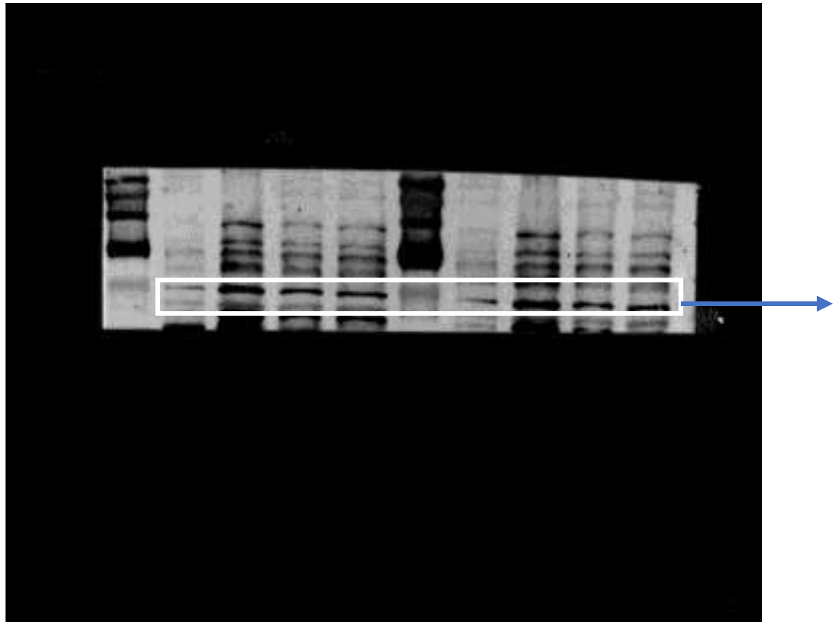

ACSL4 (79 kDa)

The labeled places represent ACSL4 in the western blot analysis of Figure 2

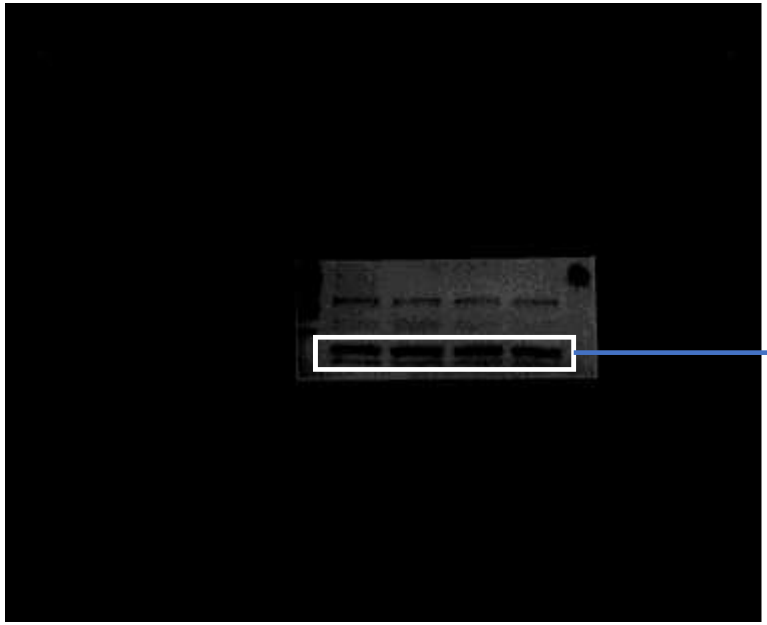

AKT (60 kDa)

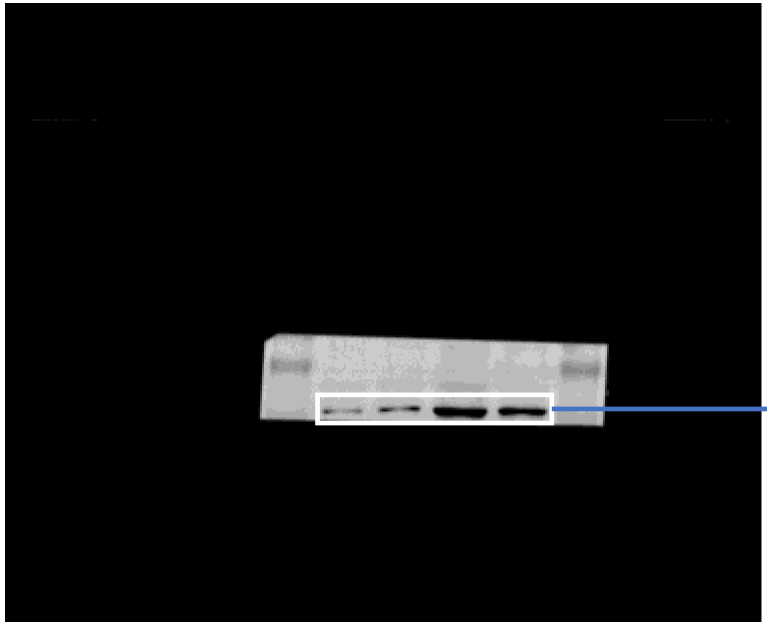

p-AKT(60 kDa)

The labeled places represent p-AKT/AKT in the western blot analysis of Figure 4

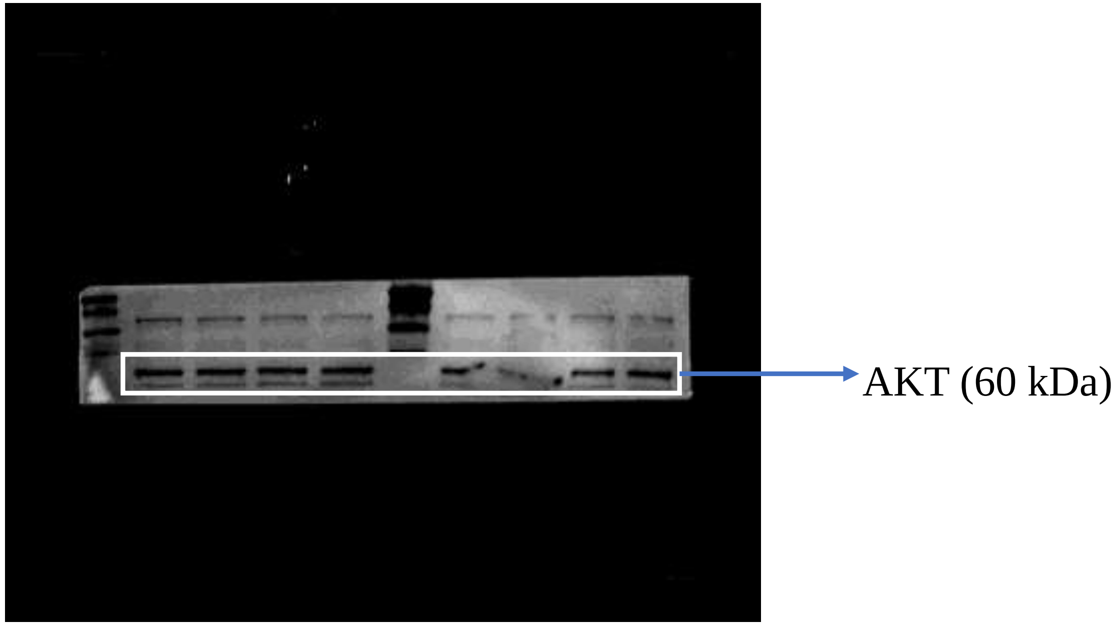
AKT (60 kDa)

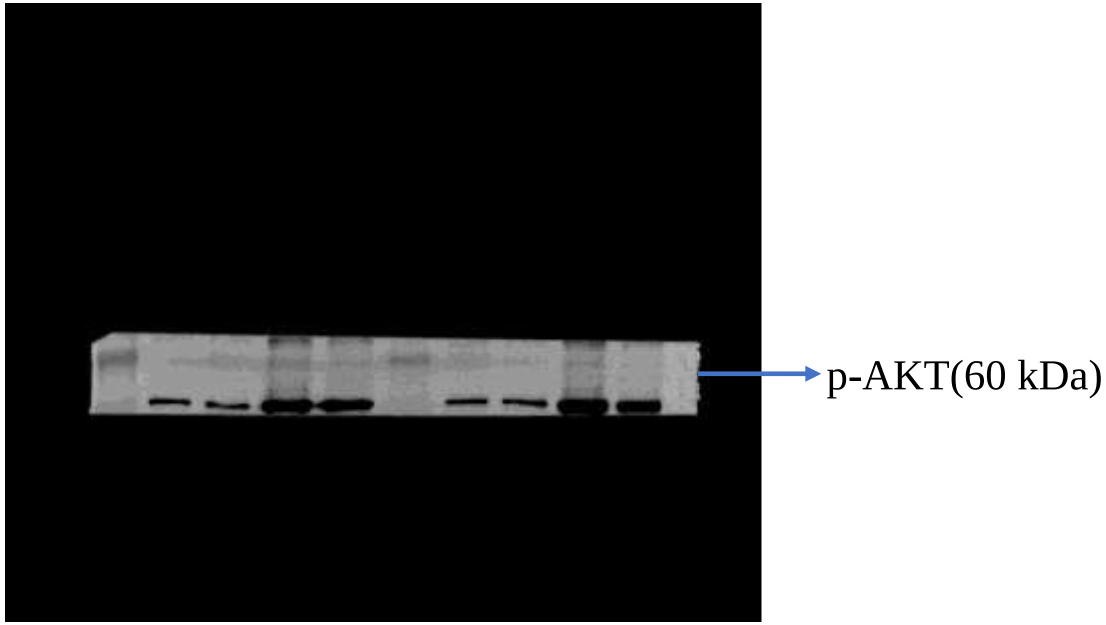
p-AKT(60 kDa)

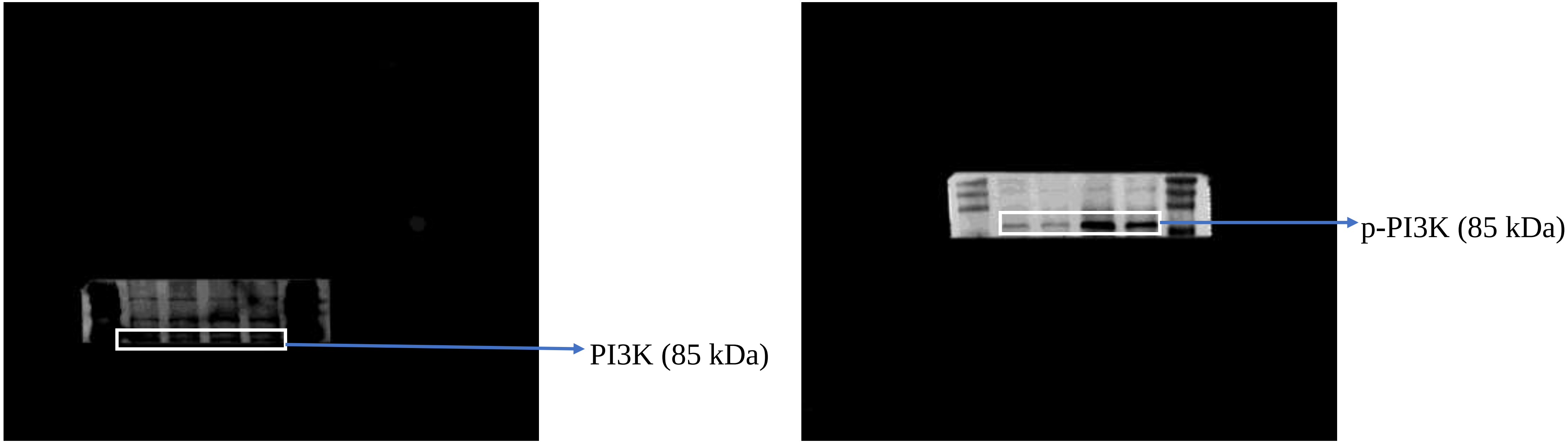

The labeled places represent p-PI3K/PI3K in the western blot analysis of Figure 4

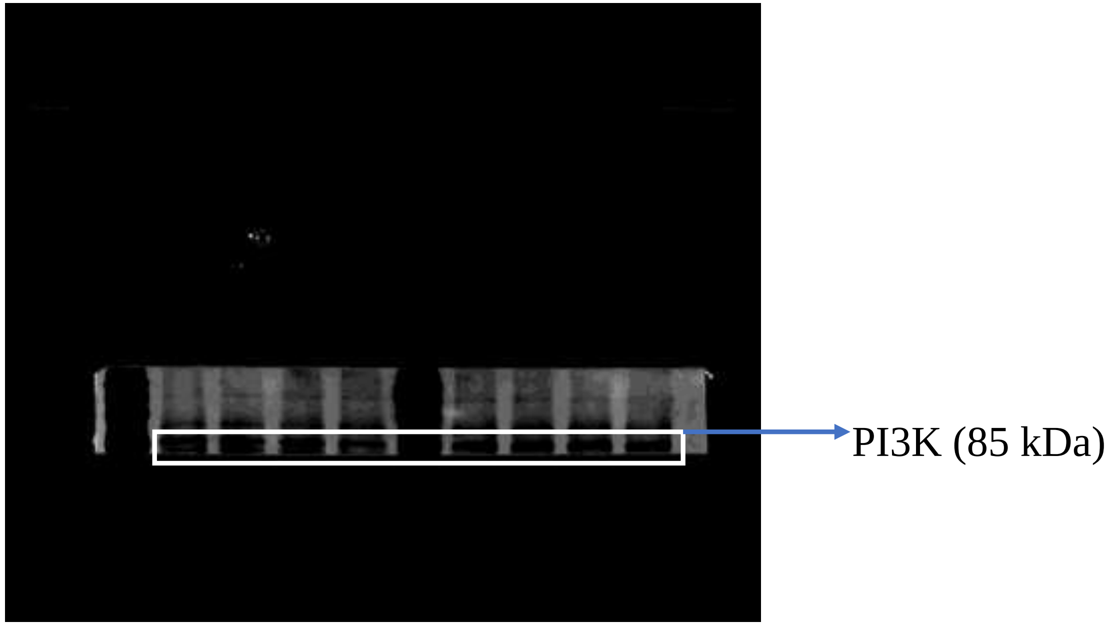
PI3K (85 kDa)

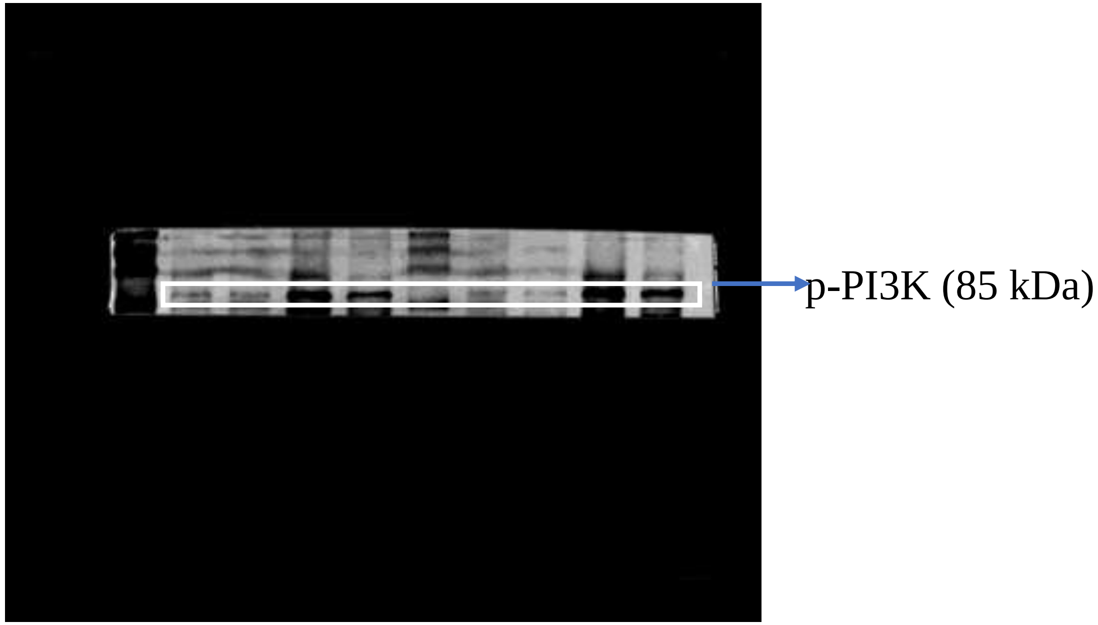
p-PI3K (85 kDa)
