# Supplementary material for: Zhilong Huoxue Tongyu capsule improves myocardial ischemia/reperfusion injury via the PI3K/AKT/Nrf2 axis
Source: PLoS One. 2024 Apr 30;19(4):e0302650. doi: 10.1371/journal.pone.0302650 (PMC11060539; doi:10.1371/journal.pone.0302650)
Supplement: S1 Table — (DOCX) [file pone.0302650.s003.docx]

Table 1. Material and reagents

| **REAGENT** | **VENDOR** | **CATALOG NO.** |
| --- | --- | --- |
| ACSL4 | Affinity | DF12141 |
| AKT | Affinity | AF4718 |
| GPX4 | Abcam | ab125066 |
| GAPDH | Affinity | AF7021 |
| HO-1 | Abcam | ab189491 |
| HRP | Affinity | S0001 |
| Nrf2 | Affinity | AF0639 |
| PI3K | Affinity | AF6241 |
| p-AKT | Abcam | ab192623 |
| p-AKT | Affinity | AF0016 |
| p- PI3K | CST | 4228T |
| p- PI3K | Zenbio | 341468 |
