# Supplementary material for: Zhilong Huoxue Tongyu capsule improves myocardial ischemia/reperfusion injury via the PI3K/AKT/Nrf2 axis
Source: PLoS One. 2024 Apr 30;19(4):e0302650. doi: 10.1371/journal.pone.0302650 (PMC11060539; doi:10.1371/journal.pone.0302650)
Supplement: S2 Table — (DOCX) [file pone.0302650.s004.docx]

Table 1. Primers used for RT-PCR

|  | **Forward (5′–3′)** | **Reverse (5′–3′)** |
| --- | --- | --- |
| β-actin | GGCTGTATTCCCCTCCATCG | CCAGTTGGTAACAATGCCATGT |
| Nrf2 | CAGCATAGAGCAGGACATGGAG | GAACAGCGGTAGTATCAGCCAG |
| HO-1 | CACTCTGGAGATGACACCTGAG | GTGTTCCTCTGTCAGCATCACC |
| GPX4 | CCTCTGCTGCAAGAGCCTCCC | CTTATCCAGGCAGACCATGTGC |
| ACSL4 | CCTTTGGCTCATGTGCTGGAAC | GCCATAAGTGTGGGTTTCAGTAC |
